# circFAM120B functions as a tumor suppressor in esophageal squamous cell carcinoma via the miR-661/PPM1L axis and the PKR/p38 MAPK/EMT pathway

**DOI:** 10.1038/s41419-022-04818-5

**Published:** 2022-04-18

**Authors:** Huan Song, Dan Tian, Jian Sun, Xuhua Mao, Weimin Kong, Dian Xu, Ye Ji, Beibei Qiu, Mengyao Zhan, Jianming Wang

**Affiliations:** 1 Department of Epidemiology, Center for Global Health, School of Public Health, Nanjing Medical University, Nanjing, 211166 China; 2 Department of Thoracic Surgery, The First People’s Hospital of Yancheng and Yancheng Clinical College of Xuzhou Medical University, Yancheng, 224001 China; 3 Department of Clinical Laboratory, Yixing People’s Hospital, Wuxi, 214200 China; 4 Department of Epidemiology, Gusu School, Nanjing Medical University, Nanjing, 211166 China

**Keywords:** Cancer epidemiology, Cancer epigenetics, Oesophageal cancer, Targeted therapies, Small RNAs

## Abstract

Extensive changes of circRNA expression underscore their essential contributions to multiple hallmarks of cancers; however, their functions and mechanisms of action in esophageal squamous cell carcinoma (ESCC) remain undetermined. Here, we adopted a three-stage approach by first screening for significantly differentially expressed circRNAs in ESCC and performing an external validation study, followed by the functional analyses. The properties of circRNAs were evaluated using Sanger sequencing, RNase R digestion, actinomycin D treatment, subcellular localization analysis, and fluorescence in situ hybridization. Target transcripts were predicted using online tools and verified by dual-luciferase, RNA immunoprecipitation, qRT-PCR, and western blot. Biotin-labeled RNA-protein pull-down, mass spectrometry, and RNA immunoprecipitation were employed to identify proteins interacting with circRNAs. Gain- and loss-of-function experiments were performed to uncover the roles of circRNAs, their target genes, and binding proteins in the proliferation, metastasis, and invasion. We observed that circFAM120B (hsa_circ_0001666) was frequently downregulated in cancer tissues and patient plasma, and its expression level was related to overall survival in ESCC patients. Overexpression of circFAM120B inhibited the proliferation, metastasis, and invasion of ESCC while silencing it enhanced malignant phenotypes. Mechanistically, circFAM120B was predominantly located in the cytoplasm, guarantying its sponging for miR-661 to restore the expression of PPM1L, a tumor suppressor. We observed that circFAM120B could reduce the stability of RNA-dependent protein kinase (PKR) by promoting its ubiquitination-dependent degradation and subsequently regulating the p38 MAPK signaling pathway, resulting in the repression of EMTs in ESCC cells. Our findings suggest that circFAM120B is a promising biomarker of ESCC, which acts as a tumor suppressor via the circFAM120B/miR-661/PPM1L axis and PKR/p38 MAPK/EMT pathway, supporting its significance as a candidate therapeutic target.

## Introduction

Esophageal carcinoma is one of the most lethal cancers, with morbidity and mortality ranking among the top ten globally [1]. Esophageal carcinomas comprise two histological subtypes, i.e., esophageal squamous cell carcinoma (ESCC) and esophageal adenocarcinoma (EAC), with the former being the predominant subtype. ESCC, which frequently occurs in the middle or upper part of the esophagus, is the dominant histological subtype in East Asia, particularly in China [2]. Although remarkable advances have been made in surgical resection, radiotherapy, and chemotherapy, the prognosis of affected patients remains unsatisfactory. The overall 5-year survival rate is less than 30%, and this is primarily ascribed to a lack of overt clinical symptoms during the early stage and a scarcity of effective screening strategies [3, 4]. Thus, yielding a non-invasive, sensitive, and specific biomarker, as well as dissecting molecular mechanisms that govern the tumorigenesis and progression of ESCC, is of profound clinical significance.

The pathogenesis of ESCC involves multiple modifiable risk factors (such as alcohol consumption, tobacco use, consuming scalding hot beverages and pickled foods, indoor air pollution, and polluted water sources) [2, 5] and heritable variations (genetic and epigenetic changes) [2, 6]. Recently, increasing evidence has demonstrated that noncoding RNAs (ncRNAs) function as epigenetic regulators in human malignancies. Circular RNA (circRNA), a novel endogenous ncRNA, has become the topic of an essential and diverse field of biological study and cancer research [7]. Although the term circRNA has been around in the literature since the last century, its impacts on regulating gene expression have not been recognized until the 2010s. With the advances in deep sequencing and bioinformatics technology, the functions of circRNAs in human health and disease have gained increasing attention. Unlike linear RNAs, circRNAs feature covalently closed continuous loop structures without a terminal 5′ cap or a 3′ poly-A tail [8]. Given its remarkable extracellular stability and evolutionary conservation, circRNAs may serve as potential biomarkers for human disorders, such as cancers [9, 10], heart failure [11], and cardiovascular diseases [12].

Previous studies have mainly focused on the vital roles of circRNAs in cancers by acting as “sponges” for microRNAs (miRNAs) and regulating RNA-binding proteins and protein translation [8, 13, 14]. Remarkably, the roles of CDR1as, which harbors almost 70 conventional binding sites for miR-7, are well established in the proliferation, apoptosis, migration, and invasion of various cancers [15–17]. Recently, much more attention has been paid to the emerging functions of circRNAs interacting with proteins. For instance, circ-HuR was found to suppress gastric cancer progression by inhibiting CNBP-induced HuR expression [18]. circECE1 could activate energy metabolism in osteosarcoma by stabilizing c-Myc expression [19]. It has been clearly demonstrated that RNA-dependent protein kinase (PKR), initially recognized as an established component of innate antiviral immunity, is crucial in multiple pathological processes, especially in viral infection and cancers [20, 21]. Cheng et al. reported that increased PKR promoted genomic instability and inferior outcomes in acute myeloid leukemia [22]. The protein kinase PKR is required to activate the p38 mitogen-activated protein kinase (MAPK) signaling pathway [23]. However, the mechanism of PKR dysregulation remains to be elucidated.

The present study adopted a three-stage design. We first screened for differentially expressed circRNAs in ESCC by integrating our previous array data and those in the publicly available Gene Expression Omnibus (GEO) database. We then performed a validation study, followed by functional analyses. We discovered that hsa_circ_0001666, designated as circFAM120B, was frequently downregulated in ESCC, and its expression was positively related to the overall survival. circFAM120B could inhibit the proliferation, migration, and invasion of ESCC by sponging miR-661 to restore PPM1L expression or destabilizing PKR to regulate the p38/EMT signaling pathway. Our findings indicate that circFAM120B might act as a promising tumor suppressor in the tumorigenesis of ESCC.

## Results

### circRNA profiling identifies circFAM120B as a candidate ESCC suppressor

**Fig. 1:**
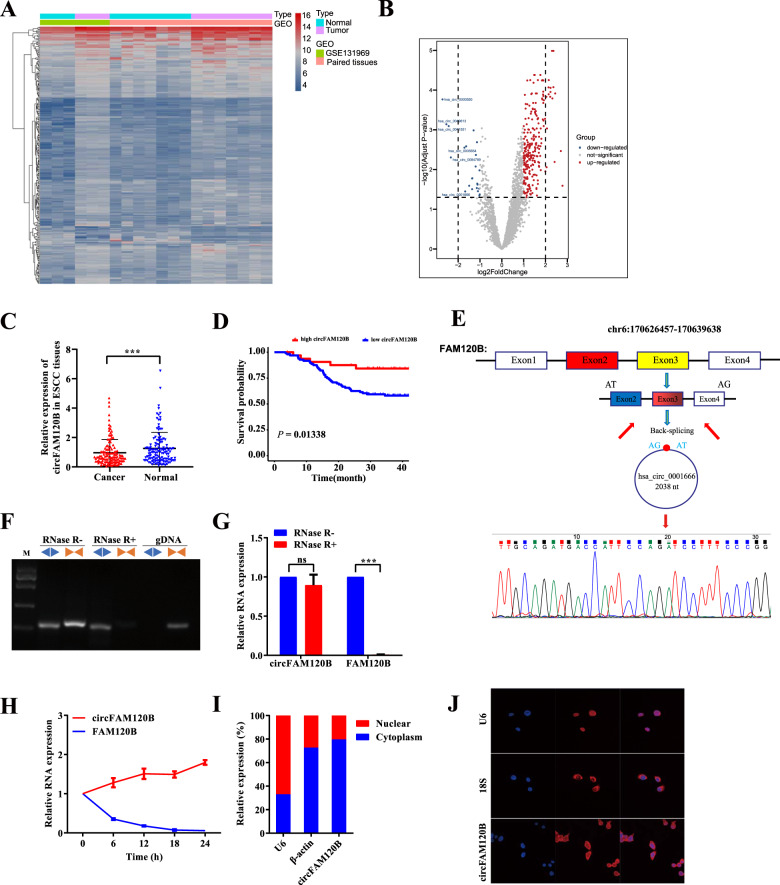
Identification of circFAM120B as a circRNA in association with ESCC. **A**, **B** The heatmap and volcano plot showed 276 differentially expressed circRNAs in ten paired ESCC tissues by Arraystar Human Circular RNA Microarray. The cutoff value was |log_2_(FC)| > 1 & *P*_adjust_ < 0.05. **C** Detection of circFAM120B in an additional 130 paired ESCC tissues by qRT-PCR analysis. **D** Kaplan–Meier analysis of ESCC patients based on circFAM120B expression (*n* = 130). The upper quartile level of circFAM120B was defined as the cutoff value. A log-rank test determined statistical significance. **E** The back-splice junction site of circFAM120B was confirmed by Sanger sequencing. **F**, **G** circFAM120B in cDNA and genomic DNA were analyzed by PCR and 2% agarose gel electrophoresis using divergent primers or convergent primers, respectively. **H** Changes in the abundance of circFAM120B and FAM120B were analyzed after treatment with actinomycin D (5 μg/ml) at indicated time points. **I** Relative abundance of circFAM120B, β-actin, and U6 in the nuclear and cytoplasmic fractions of KYSE-150 cells was analyzed. **J** circFAM120B localization was analyzed by RNA FISH in KYSE-150 cells. ***P < 0.001.

We performed a screening assay in seven pairs of ESCC and adjacent normal-appearing tissues using a circRNA microarray [10]. Additionally, we interrogated the Gene Expression Omnibus (GEO) database and selected GSE131969 as a candidate dataset. With an integrated analysis, we identified 276 distinct circRNAs (FC > 1 & *P*_adjust_ < 0.05), of which 253 were upregulated, and 23 were downregulated in cancerous tissues (Fig. 1A, B). To validate in silico discovery, we filtered for circRNAs longer than 2500 nucleotides (nts) and verified the top ten downregulated circRNAs in 10 pairs of ESCC tissues. The characteristics of these circRNAs are detailed in Supplementary Table 1. Of these, circFAM120B was the most significantly downregulated. This finding was confirmed in 130 other pairs of ESCC tissues (Fig. 1C). We further explored the correlation between circFAM120B levels and clinicopathological characteristics and found that circFAM120B expression was negatively associated with tumor size (Supplementary Table 2). Patients with low expression of circFAM120B had a poor prognosis (Fig. 1D). These data indicated that circFAM120B was frequently downregulated in ESCC and negatively associated with malignant features.

### Characterization of circFAM120B and its expression in ESCC

circFAM120B (chr6: 170,626,457-170,639,638) has 2038 nts and originates from the 2, 3 and 4 exons of FAM120B. The back-splice junction site of circFAM120B was amplified using divergent primers and confirmed by Sanger sequencing (Fig. 1E). PCR analysis showed that circFAM120B could be amplified by divergent primers from cDNA but not genomic DNA (Fig. 1F). Resistance to RNase R exonuclease digestion confirmed that circFAM120B existed as a closed-loop structure (Fig. 1F, G). Treatment with actinomycin D similarly showed that circFAM120B was more stable than FAM120B mRNA (Fig. 1H). After fractionating KYSE-150 cells into nuclear and cytoplasmic lysates, the qRT-PCR analysis revealed that circFAM120B was predominantly located in the cytoplasm (Fig. 1I). In line with the observations in fractionated lysates of KYSE-150 cells, FISH examination confirmed that circFAM120B was less abundant in the nucleus when 18S and U6 were used as markers for the cytoplasm and nucleus, respectively (Fig. 1J). These results suggested that circFAM120B was a genuine circular RNA primarily located in the cytoplasm.

### circFAM120B inhibits tumorigenicity of ESCC in vitro

We generated cells stably overexpressing circFAM120B and confirmed that circFAM120B was successfully overexpressed in ESCC cells but did not alter FAM120B mRNA levels (Supplementary Fig. 1A, B). To knock down circFAM120B, we employed siRNAs that specifically targeted its back-splice junction region. Of these, si-circFAM120B#2 and si-circFAM120B#3 were confirmed to silence the expression of circFAM120B but not affect the FAM120B mRNA expression in ESCC cells and were then selected for subsequent functional analyses (Supplementary Fig. 1A, B).

**Fig. 2:**
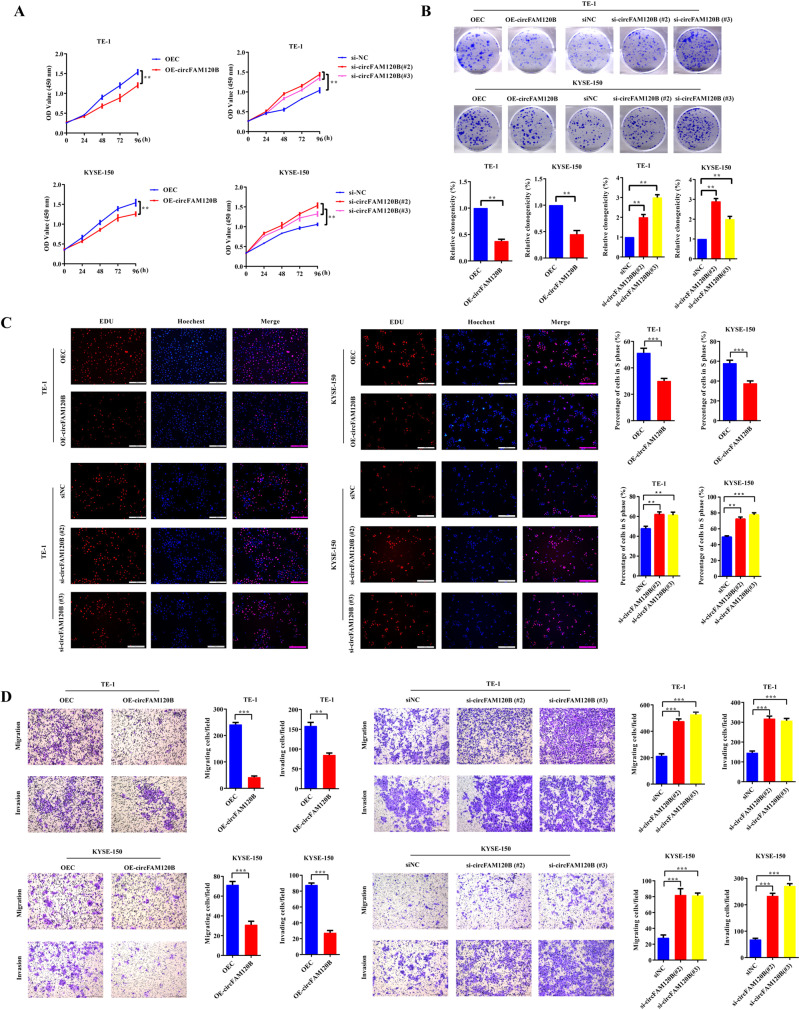
circFAM120B acts as a tumor suppressor in ESCC cells. **A** Proliferation of ESCC cells with circFAM120B overexpression or knockdown was evaluated by CCK-8 assay (*n* = 5 biologically independent replicates). **B** Colony formation assays were performed in ESCC cells with circFAM120B overexpression or knockdown (*n* = 3 biologically independent replicates). **C** Detection of proliferating ESCC cells with circFAM120B overexpression or knockdown by EdU assay (*n* = 3 biologically independent replicates). **D** Migration and invasion of ESCC cells with circFAM120B overexpression or knockdown were assessed by Transwell assays (*n* = 3 biologically independent replicates). **P < 0.01, ***P < 0.001.

Cell viability was assessed by CCK-8, colony formation, and EdU assays. Overexpression of circFAM120B caused a prominent decrease in cell proliferation rates, whereas circFAM120B silencing led to a striking increase (Fig. 2A–C). Transwell assays showed that the migration and invasion of ESCC cells were remarkably suppressed by circFAM120B overexpression and aggravated by circFAM120B siRNAs (Fig. 2D). These findings imply the antitumor functions of circFAM120B in ESCC in vitro.

### circFAM120B serves as a miRNA sponge for miR-661 in ESCC cells

**Fig. 3:**
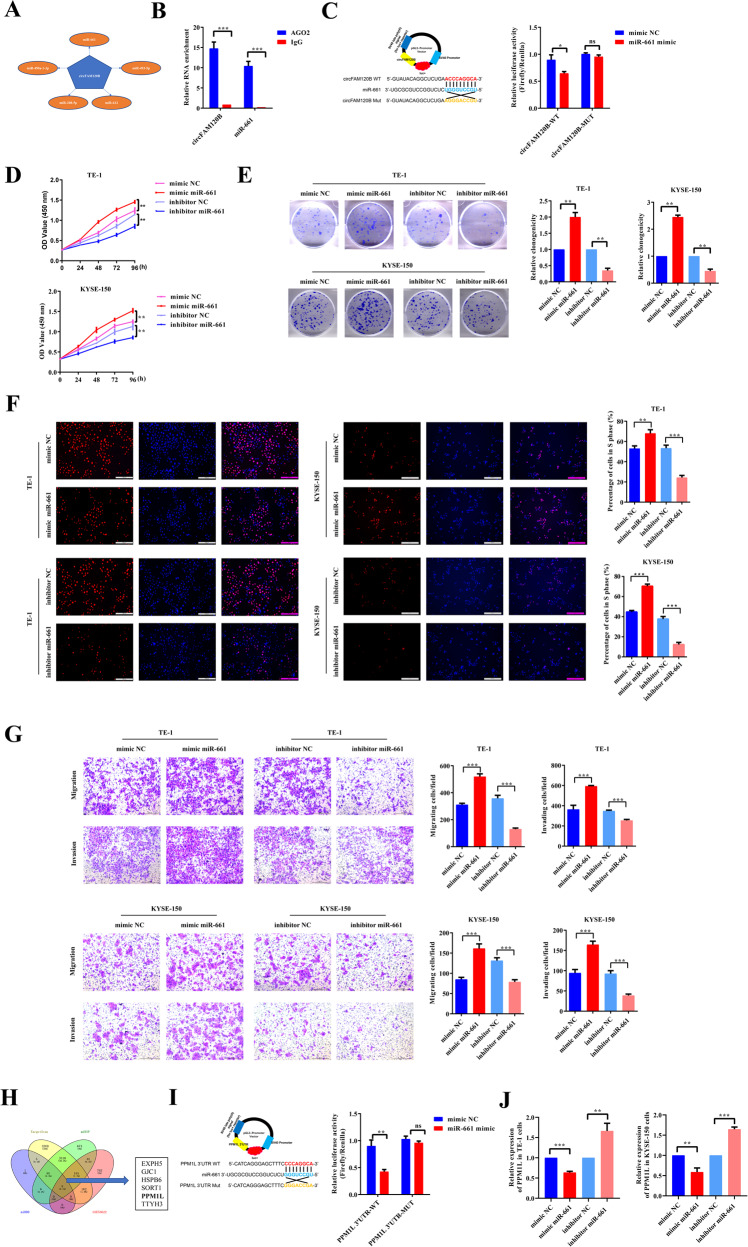
miR-661 enhances tumorigenicity of ESCC by reducing PPM1L expression. **A** The top five miRNAs as microRNA response elements for circFAM120B. **B** RIP assays using an anti-AGO2 antibody, followed by qRT-PCR analysis, confirm the interactions between circFAM120B and miR-661. **C** A schematic representation of the 3’-UTR of circFAM120B with the predicted target site for miR-661, as well as the mutant sites of circFAM120B. Luciferase reporter analysis was performed to evaluate the binding between miR-661 and circFAM120B. Reporter constructs containing either circFAM120B-wt or circFAM120B-mut were co-transfected into HEK293T cells, along with miR-661 or miR-NC mimics. **D** The proliferation of ESCC cells transfected with miR-661 mimics or inhibitors was evaluated by CCK-8 assay (*n* = 5 biologically independent replicates). **E** Colony formation assays were performed in ESCC cells transfected with miR-661 mimics or inhibitors (*n* = 3 biologically independent replicates). **F** Analysis of proliferating ESCC cells transfected with miR-661 mimics or inhibitors by EdU assay (*n* = 3 biologically independent replicates). **G** Migration and invasion of ESCC cells transfected with miR-661 mimics or inhibitors were evaluated by Transwell assays (*n* = 3 biologically independent replicates). **H** Venn diagram showing the potential mRNAs targeted by miR-661. **I** A schematic representation of the 3’-UTR of PPM1L with the predicted target site for miR-661 and the mutant sites of PPM1L. Luciferase reporter analysis was performed to evaluate the binding between miR-661 and PPM1L. Reporter constructs containing either PPM1L-wt or PPM1L-mut were co-transfected into HEK293T cells, along with miR-661 mimics or miR-NC. **J** Expression of PPM1L was assessed by qRT-PCR in ESCC cells transfected with miR-661 mimics or inhibitors. *P < 0.05, **P < 0.01, ***P < 0.001.

circRNAs in the cytoplasm may function as miRNA sponges, thereby abrogating the inhibitory impact of miRNAs on target mRNAs [15]. Given the primarily cytoplasmic distribution and superior stability of circFAM120B, it is conceivable that it functions as a ceRNA in ESCC progression. Therefore, we used the circular RNA interactome (CircInteractome, https://circinteractome.nia.nih.gov/) database and Arraystar (TargetScan: http://www.targetscan.org/; miRanda: http://www.microrna.org/) to predict potential circFAM120B-miRNA interactions. circFAM120B possessed a conserved target site for miR-661 with the highest scores (Fig. 3A). Argonaute2 (AGO2), a vital component of the RNA-induced silencing complex (RISC), can mediate the circRNA-miRNA interaction [24]. To determine whether miR-661 binds to circFAM120B, we performed an RNA RIP assay with an AGO2 antibody. The results indicated that circFAM120B and miR-661 were efficiently enriched by the AGO2 antibody (Fig. 3B) compared with the IgG control, suggesting that circFAM120B could directly interact with miR-661. WT-circFAM120B co-transfection significantly reduced the luciferase activity, but MUT-circFAM120B failed to exert the same effect (Fig. 3C). These experiments collectively demonstrated that circFAM120B could act as a sponge for miR-661 in ESCC cells.

### miR-661 enhances the tumorigenicity of ESCC by depleting PPM1L in vitro

We further investigated the biological functions of miR-661 in ESCC cells. As expected, miR-661 mimics significantly enhanced the proliferation of ESCC cells, as indicated in CCK-8, colony formation, and EdU assays, while miR-661 inhibitors remarkably inhibited it (Fig. 3D–F). Transwell assays showed that miR-661 mimics promoted the migration and invasion of ESCC cells, whereas miR-661 inhibitors restrained them (Fig. 3G). Hence, we concluded that miR-661 had a positive effect on the malignant phenotypes of ESCC.

**Fig. 4:**
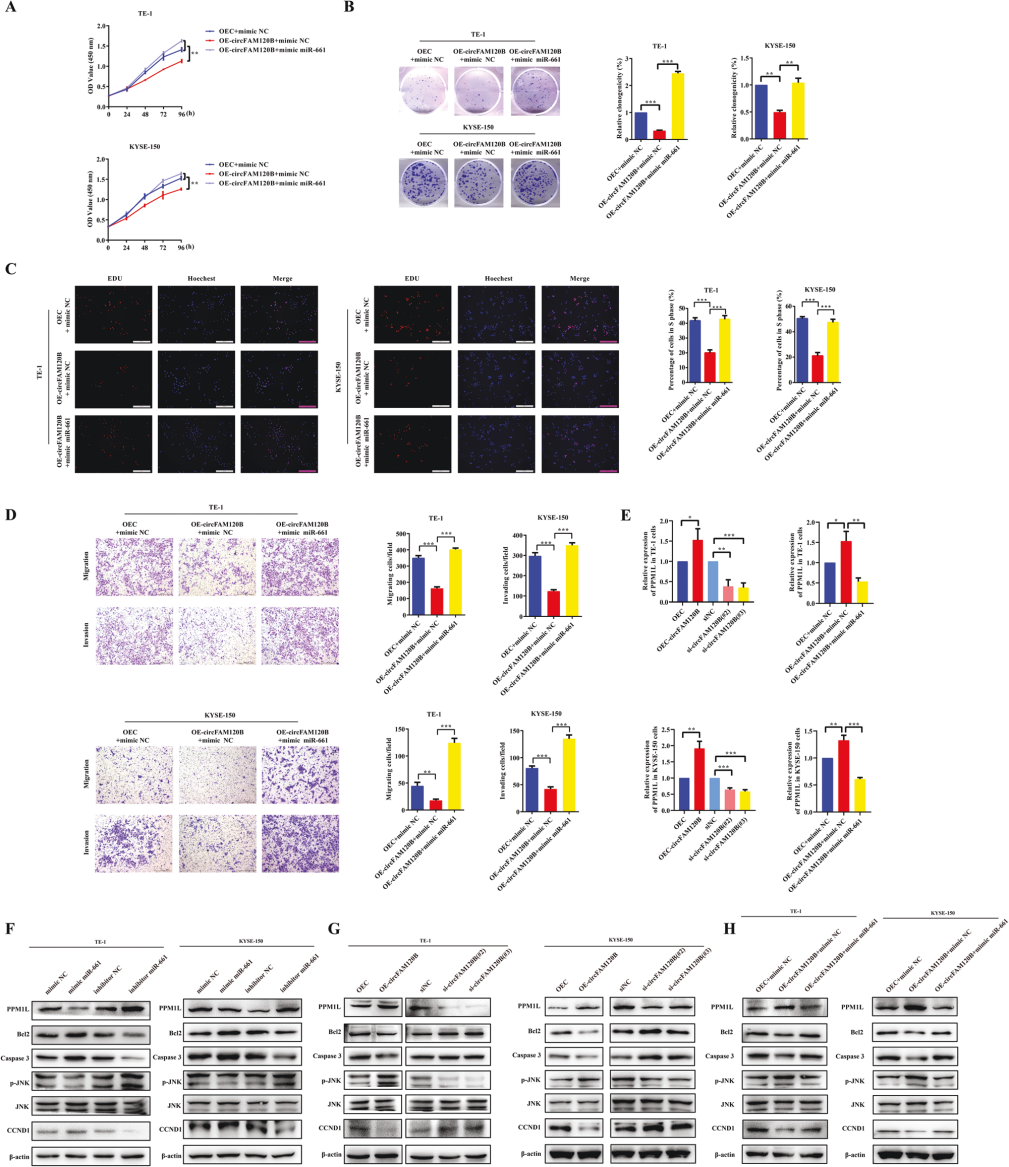
circFAM120B inhibits the tumorigenicity of ESCC by binding with miR-661 to restore PPM1L expression. **A** Proliferation of ESCC cells co-transfected as indicated was assessed by CCK-8 assay (*n* = 5 biologically independent replicates). **B** Colony formation assays were performed in ESCC cells co-transfected as indicated (*n* = 3 biologically independent replicates). **C** Detection of the proliferating ESCC cells with circFAM120B overexpression or knockdown by EdU assay (*n* = 3 biologically independent replicates). **D** Migration and invasion of ESCC cells co-transfected as indicated were examined by Transwell assays (*n* = 3 biologically independent replicates). **E** Expression of PPM1L was examined by qRT-PCR in ESCC cells transfected as indicated. **F**–**H** The expression levels of specific proteins were analyzed by western blot analysis in ESCC cells treated as indicated. *P < 0.05, **P < 0.01, ***P < 0.001.

We searched three online tools for potential target genes of miR-661, including TargetScan (http://www.targetscan.org/mamm_31/), miRDB (http://mirdb.org/, score ≥85), and mirDIP (http://ophid.utoronto.ca/mirDIP/, scores were very high or high). After identifying downregulated mRNAs in GSE53622 with the criteria of log_2_FC ≥ 1.5 & FDR < 0.05, we intersected these four datasets and identified eight candidates (TTYH3, PLEKHA6, HSPB6, SORT1, ATOH8, GJC1, PPM1L, EXPH5). Of these, PPM1L, which is involved in cell apoptosis, attracted our interest (Fig. 3H). Overexpression of PPM1L inhibited the proliferation, migration, and invasion of ESCC, whereas silencing it promoted these malignant phenotypes (Supplementary Figs. 1 and 2). A subsequent luciferase reporter assay revealed that co-transfection of miR-661 mimics and WT-PPM1L-3′ UTR decreased luciferase activity, while the MUT-PPM1L-3′ UTR exerted no such effect (Fig. 3I). As expected, both the mRNA and protein levels of PPM1L were reduced by miR-661 mimics but enhanced by miR-661 inhibitors (Figs. 3J and 4F). Overall, miR-661 may promote the tumorigenicity of ESCC by depleting PPM1L.

### miR-661 reduces the tumor-suppressive capacity of circFAM120B via PPM1L in vitro

A series of rescue experiments were designed to elucidate whether circFAM120B regulates tumor progression via this newly identified circFAM120B/miR-661/PPM1L axis. Rescue experiments demonstrated that miR-661 mimics could effectively reverse the inhibition of the proliferation, migration, and invasion induced by circFAM120B overexpression in CCK-8, colony formation, and EdU assays (Fig. 4A–D). Overexpression of circFAM120B remarkably increased the expression of PPM1L while silencing circFAM120B markedly decreased it (Fig. 4E, G). The altered expression of PPM1L induced by circFAM120B modulation could be reversed by miR-661 mimics (Fig. 4E, H). These data showed that circFAM120B could sponge miR-661 to restore PPM1L expression.

To explore whether circFAM120B could regulate proliferation-associated marker proteins and participate in the progression of ESCC, we examined the proteins expression of Bcl2, Caspase3, p-JNK, JNK, and CCND1. miR-661 mimics markedly elevated the expression of Bcl2, Caspase3, and CCND1 while reducing the levels of p-JNK. miR-661 inhibitors exerted opposite effects (Fig. 4F). Conversely, overexpression of circFAM120B markedly suppressed Bcl2, Caspase3, and CCND1, while enhancing the levels of p-JNK. circFAM120B knockdown exerted opposite effects (Fig. 4G), and miR-661 mimics reversed these changes induced by circFAM120B (Fig. 4H).

### circFAM120B binds to PKR and promotes its polyubiquitination and degradation

**Fig. 5:**
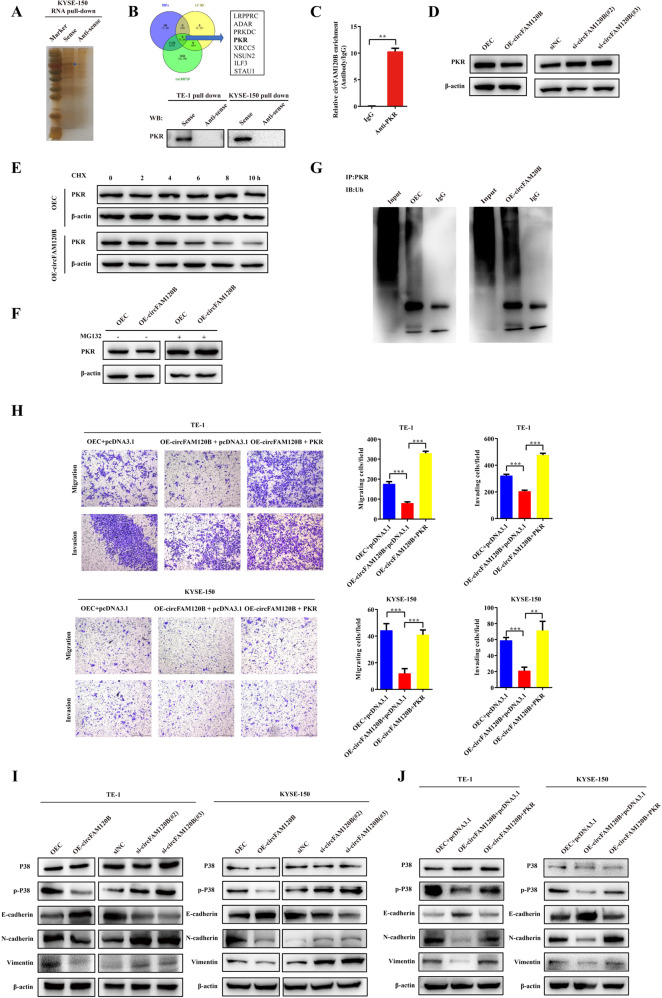
circFAM120B physically interacts with PKR and promotes its ubiquitin/proteasome-mediated degradation. **A** Silver staining of proteins pulled down by circFAM120B. **B** A total of 8 candidate proteins were identified after the intersection of the RNA pull-down dataset (peptides >5), website predictions (catRAPID, http://service.tartaglialab.com/page/catrapid_omics2_group), and classic RNA binding protein datasets. The specific amino acid sequences were detected by mass spectrometry. PKR was pulled down by a circFAM120B sense RNA probe but not by the antisense probe. **C** RIP assays with qRT-PCR show that circFAM120B was pulled down by an anti-PKR antibody in KYSE-150 cells. **D** The specific association of PKR and circFAM120B was detected by western blot analysis. **E** KYSE-150 cells stably overexpressing circFAM120B or controls were treated with cycloheximide (CHX, 50 µg/ml) at indicated time points and analyzed by western blot. **F** KYSE-150 cells stably overexpressing circFAM120B or controls were treated with MG132 (25 mmol/L) for 10 h and analyzed by western blot. β-actin was used as an internal control. **G** The ubiquitination of PKR was detected by western blot in KYSE-150 cells with or without circFAM120B overexpression. **H** Rescue experiments indicated that PKR was essential for circFAM120B-induced inhibition of migration and invasion. **I**, **J** Protein levels were evaluated by western blot assays in ESCC cells with the indicated treatments. **P < 0.01, ***P < 0.001.

Based on insights gained from the roles of other ncRNAs as protein interaction partners, we reasoned that circFAM120B might directly interact with proteins and modulate the function of RNA-binding proteins. We pulled down proteins with biotinylated circFAM120B and then analyzed them by mass spectrometry. A total of 8 candidate proteins were identified after the intersection of the RNA pull-down dataset (peptides >5), website predictions (catRAPID), and classic RNA binding protein datasets (Fig. 5A, B). Here, we were especially interested in the PKR, a serine/threonine-protein kinase that could be activated by binding to dsRNA, and chose it as a candidate circFAM120B-associated protein. Next, the binding of PKR to circFAM120B was confirmed by western blot and RIP assays with an anti-PKR antibody (Fig. 5B, C). We found that circFAM120B did not affect PKR mRNA expression, whereas overexpression of circFAM120B significantly reduced the PKR protein levels, and that silencing of circFAM120B increased its stability (Supplementary Fig. 1D and Fig. 5D). Overexpression of circFAM120B reduced the half-life of the PKR protein (Fig. 5E). However, in the presence of the proteasome inhibitor MG132, overexpression of circFAM120B no longer promoted the degradation of PKR, suggesting that circFAM120B could accelerate the proteasome-dependent degradation of PKR (Fig. 5F). Moreover, PKR became significantly ubiquitinated after overexpression of circFAM120B (Fig. 5G). Collectively, these observations demonstrated that circFAM120B reduced the stability of the PKR protein by promoting its ubiquitin/proteasome-dependent degradation.

### PKR is a functional mediator of the circFAM120B-regulated p38 MAPK/EMT pathway

Subsequently, we reviewed the UALCAN online tool (http://ualcan.path.uab.edu/cgi-bin/ualcan-res.pl) and found that PKR expression was upregulated in ESCC tissues (Supplementary Fig. 3), indicating the carcinogenic effects of PKR in ESCC. In view of the essential role of PKR in the p38 MAPK signaling pathway [25], as well as the roles of p38 MAPK in EMT [26], we hypothesized that circFAM120B might also exert its EMT progress on ESCC through the PKR-mediated p38 MAPK/EMT pathway. We first evaluated the effects of PKR on circFAM120B-induced migration and invasion inhibition. As shown in Fig. 5H, ectopic expression of PKR abolished circFAM120B-induced phenotypic inhibitions. We found that circFAM120B did not affect p38 protein levels but negatively regulated its phosphorylation, as well as the protein levels of N-cadherin and vimentin while positively regulating E-cadherin proteins (Fig. 5I). Moreover, PKR reversed the circFAM120B-induced changes of these proteins (Fig. 5J and Supplementary Fig. 4A, B). For further confirmation, we knocked down circFAM120B in ESCC cells, followed by p38 MAPK inhibitor SB 203580 treatment. We observed that the enhanced phosphorylation of p38 and overexpression of N-cadherin and Vimentin by circFAM120B knockdown was weakened by SB 203580 treatment (Supplementary Fig. 4C). These results indicated that PKR was a functional mediator of circFAM120B-dependent regulation of the p38 MAPK signaling pathway and thus affected the EMT of ESCC cells.

### circFAM120B attenuates tumorigenesis and metastasis of ESCC cells in vivo

**Fig. 6:**
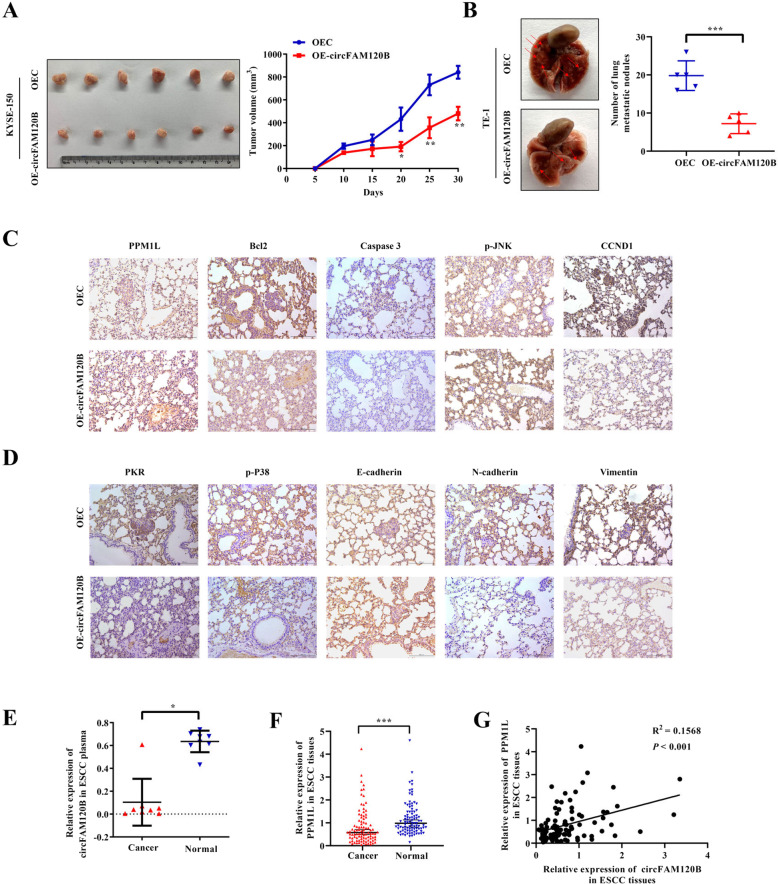
circFAM120B depresses the tumorigenicity of ESCC in vivo and is associated with PPM1L in clinical samples. **A** KYSE-150 cells with indicated modifications were subcutaneously injected into the flanks of mice in the armpit. Subcutaneous xenograft tumors, as well as changes in tumor size, are displayed. **B** TE-1 cells with indicated modifications were subcutaneously injected from the tail vein of nude mice. The lung tissues of the mice were harvested, and the number of metastatic foci was counted. **C**, **D** Indicated proteins were detected by IHC assays in murine tissues. The representative images are presented. **E** Detection of circFAM120B expression in eight pairs of plasma samples from ESCC patients and healthy volunteers by qRT-PCR analysis. **F** Assessment of PPM1L expression in 96 pairs of ESCC tissues by qRT-PCR analysis. **G** Correlation between circFAM120B and PPM1L expression in 96 pairs of ESCC tissues.

To further elucidate the biological functions of circFAM120B in vivo, we established mouse models of xenograft tumor growth and lung metastasis. We found that the xenograft tumors of circFAM120B-overexpressed KYSE-150 cells were significantly smaller in volume than those of negative controls (Fig. 6A). Moreover, circFAM120B overexpression resulted in fewer lung metastatic lesions (Fig. 6B). Next, the abundance of PPM1L, Bcl2, Caspase 3, p-JNK, CCND1, PKR, p-P38, E-cadherin, N-cadherin, and vimentin was assessed by immunohistochemical staining, and these results were consistent with the results of in vitro experiments (Fig. 6C, D).

### Clinical implications of circFAM120B in patients with ESCC

To determine whether circFAM120B serves as a “liquid biopsy” biomarker for ESCC, we compared the abundance of circFAM120B in plasma between healthy volunteers and preoperative plasma from patients with ESCC. We found that the abundance of circFAM120B in preoperative plasma was lower than that in healthy volunteers (Fig. 6E). To further verify the clinical relevance of circFAM120B and PPM1L in ESCC, we examined their levels in 96 ESCC patients and found that the expression of PPM1L was lower in ESCC tissues than in adjacent normal-appearing tissues in ESCC (Fig. 6F). The expression of PPM1L was positively correlated with the levels of circFAM120B (Fig. 6G). Altogether, these findings confirmed our findings in vitro, supporting the clinical utility of circFAM120B as a biomarker for ESCC.

## Discussion

**Fig. 7:**
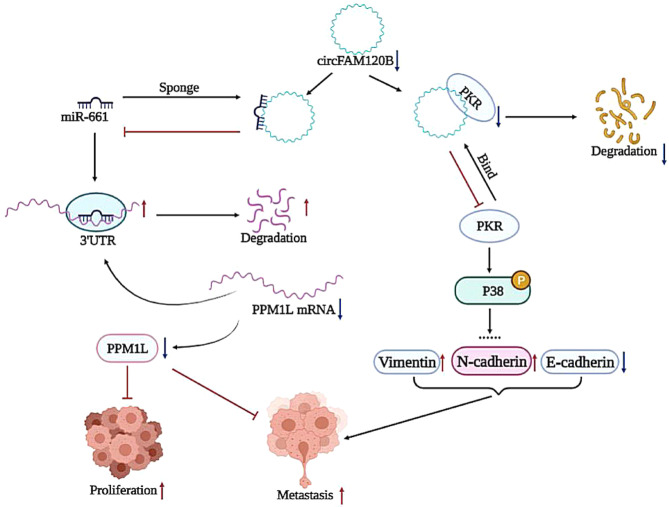
circFAM120B acts as a tumor suppressor via the circFAM120B/miR-661/PPM1L axis and PKR/p38 MAPK/EMT pathway. Schematic representation of the molecular pathway of circFAM120B-mediated tumorigenesis.

It is estimated that approximately 2% of transcripts encode proteins, while the majority are transcribed as ncRNAs in mammals [27, 28]. CircRNAs, as an emerging subgroup of ncRNAs, have recently been implicated in diverse cellular processes, especially in the proliferation, invasion, metastasis, and therapeutic resistance of tumors [29, 30]. This study identified differentially expressed circRNAs in ESCC by integrating our previous microarray data and GEO datasets and selecting potential candidates by large-scale qRT-PCR analysis. We carefully characterized a series of dysregulated circRNAs in ESCC tissues, especially the downregulated hsa_circ_0001666 (log_2_FC = −1.68). The genomic location for hsa_circ_0001666, subsequently designated as circFAM120B, is chr6: 170626457-170639638, and the spliced length is 2038 nts. Functional experiments showed that circFAM120B was a tumor suppressor in ESCC. Mechanistically, we proposed a model in which circFAM120B functioned as a ceRNA that competitively bound to miR-661 and reversed the inhibitory effect of miR-661 on its target PPM1L mRNA, thereby regulating the proliferation, migration, and invasion of ESCC. Furthermore, PKR was identified as a functional mediator of the circFAM120B-dependent regulation of the p38 MAPK signaling pathway and thus affected the EMT of ESCC cells (Fig. 7).

Unlike traditional linear RNA, circRNA is generated by back-splicing into a covalently closed loop without a 5’-cap or a 3’-poly(A) tail, which facilities its structural stability [31]. Physical properties of circFMA120B were consistent with these characteristics; circFAM120B, as a novel circular RNA, exhibited better tolerance to RNase R digestion and actinomycin D treatment than the linear FAM120B. circRNAs are characterized by high abundance and tissue- or developmental stage-specific expression patterns [32], suggesting their unique roles as biomarkers in human diseases. Emerging studies have revealed the diversity and dynamics of circRNAs in the initiation, progression, and prognosis of tumors [33], immune responses [34], and pathogen infections [35]. Accordingly, several circRNAs have been implicated in the clinicopathology of human cancers. The present molecular epidemiological study found that higher expression of circFAM120 was associated with a better prognosis and that the expression of circFAM120B in ESCC tissues was negatively related to tumor size. We also found that the expression of circFAM120B was downregulated in plasma from patients with ESCC, suggesting its potential utility as a “liquid biopsy” biomarker for ESCC. However, research on the clinical implications of circFAM120B in plasma is limited by the sample size.

Dysregulated circRNAs exert tumor-suppressive or oncogenic roles in various cancers. For example, circNDUFB2 was identified as a tumor suppressor in non-small cell lung cancer, while circ-TTBK2 exerted an oncogenic role in glioma [35, 36]. In the present study, we showed that circFAM120B functioned as a tumor suppressor in ESCC, with the capability to restrain its malignant phenotypes. Following biogenesis, most circRNAs, except for intron-containing circRNAs, are transported from the nucleus to the cytoplasm in an ATP-dependent manner [8], promoting the classical model of circRNAs as miRNA sponges. In this study, we confirmed the role of circFAM120B as a sponge for miR-661, which has been identified as an oncogenic factor in multiple cancers [35, 37]. It has been well documented that miRNAs usually suppress target mRNAs by binding to the 3′ untranslated region (3′ UTR) in a sequence-specific manner [38]. Especially, miR-661 was reported to contribute to the cell proliferation of ovarian cancer by inhibiting INPP5J expression [37]. Here, we discovered for the first time that PPM1L, a member of the protein phosphatase 2C (PP2C) superfamily, was a potential target of miR-661 in ESCC cells. Previous studies indicated that PPM1L was a tumor suppressor in colorectal tumorigenesis via negatively regulating TGF-β and BMP signaling pathways [39]. Our bioinformatics analysis based on GSE131969 and data from 96 pairs of ESCC tissues showed a downregulation trend of PPM1L, consistent with its potential function as a tumor suppressor in ESCC. Altogether, our findings demonstrated that the functions of circFAM120B are at least partially mediated through the miR-661/PPM1L axis.

Even though numerous circRNAs function as endogenous competing RNAs or miRNA sponges, this regulatory mechanism remains insufficient due to the limited miRNA binding sites. Interestingly, circRNAs also exert their biological functions by interacting with specific proteins. We identified PKR as an interacting partner of circFAM120B. PKR, a double-stranded RNA (dsRNA)-dependent protein kinase, has multiple functions in cancers, including regulating hepatocellular carcinoma tumorigenesis and sensitivity to trastuzumab therapy in breast cancer [20, 40]. It has been well established that PKR could be activated by autophosphorylation after binding to dsRNA. Activated PKR phosphorylates translation initiation factor EIF2S1, which in turn suppresses protein synthesis. Conversely, when PKR binds to E3 ligase, its ubiquitination is enhanced, which leads to degradation [41]. Interestingly, we observed, for the first time, that circFAM120B reduced the stability of the PKR protein by promoting its ubiquitination-dependent degradation. Our rescue experiments highlighted the contributions of PKR in reversing circFAM120B-mediated tumor inhibition in ESCC. Notably, PKR participates in multiple cancer-related pathways, especially the p38 MAPK signaling pathway [23]. In addition, the p38 MAKP signaling pathway is implicated in several tumors by multiple lines of evidence, including ESCC [42]. Epithelial-mesenchymal transition (EMT) enables the dissemination and distal metastasis of primary tumors. It has been widely reported that the p38 MAKP signaling pathway is frequently associated with the EMT progression in breast cancer and lung cancer [43, 44]. Yuan et al. found that gelsolin could suppress the metastasis of gastric cancer through inhibition of PKR-p38 signaling [45]. As expected, we observed the effects of PKR on circFAM120B-regulated phosphorylation of p38, thereby regulating the EMT of ESCC cells. Altogether, our findings broaden insights into the mechanisms and biological functions of circFAM120B that it functions by interacting with protein partners, as well as by modulating PKR degradation.

## Conclusion

hsa_circ_0001666, designated as circFAM120B, is frequently downregulated in ESCC and positively related to overall survival. Functionally, circFAM120B substantially inhibits the proliferation, migration, and invasion of ESCC by sponging miR-661 to restore PPM1L expression or destabilizing PKR to modulate the p38/EMT signaling pathway. Our findings indicated that circFAM120B might be a promising target of diagnosis and treatment because of its function as a tumor suppressor in ESCC.

## Materials and methods

### Patient sets and specimens processing

Human ESCC and adjacent normal-appearing tissues were collected from 130 patients who underwent an esophagectomy in the First People’s Hospital of Yancheng City between November 2016 and December 2018. We also collected 8 pairs of plasma samples from preoperative patients with ESCC and healthy volunteers at the First People’s Hospital of Yixing City in 2019, which were matched by age, sex, tobacco smoking, and alcohol consumption. None of them received chemoradiotherapy before sample collection. Patients’ clinical information is summarized in Supplementary Tables 2 and 3. The pathological tumor-node-metastasis (TNM) status was assessed according to the American Joint Committee on Cancer (AJCC) TNM staging criteria. All patients were followed annually. The overall survival (OS) was defined as the time from the surgery to death or last follow-up for survivors.

### Microarray and data analysis

We reviewed the publicly available Gene Expression Omnibus (GEO) database (https://www.ncbi.nlm.nih.gov/geo/) for circRNA expression datasets in ESCC with the following filters: (1) the dataset was derived from ESCC tissue samples; (2) the detection regimen was microarray analysis; and (3) the sample size was at least three. Finally, the GSE131969 dataset, consisting of three pairs of ESCC and adjacent non-cancerous tissues, was identified. Then, we integrated our previous circRNA datasets with GSE131969 by batch normalization using the “sva” package and subsequently applied the “limma” package to profile the dysregulated circRNAs with a filter criterion of |fold change| > 1 and false discovery rate (FDR) < 0.05.

### Cell culture and reagents

Human ESCC cell lines (KYSE-150 and TE-1) and a human embryonic kidney cell line (HEK293T) were purchased from the Institute of Biochemistry and Cell Biology of the Chinese Academy of Sciences (Shanghai, China). Cells were maintained in RPMI-1640 medium (KYSE-150 and TE-1) or DMEM (HEK293T) supplemented with 10% FBS (Biological Industries, Beit HaEmek, Israel) and 1% penicillin/streptomycin (Gibco) in a humidified incubator at 37 °C containing 5% CO_2_. All cells used in this study were authenticated by short tandem repeat (STR) DNA profiling and used for experiments within 15 generations from initial resuscitation. The routine detection confirmed that all cells were free from mycoplasma contamination. The SB203580 was employed for p38 MAPK signal pathway inhibitory (ApexBio, Texas, USA).

### RNA and genomic DNA (gDNA) extraction

Total RNA was purified from ESCC tissues and cells using TRIzol reagent (Invitrogen, Waltham, MA) and plasma using a miRNeasy Serum/Plasma Kit (Qiagen, Hilden, Germany). RNA in nuclear and cytoplasmic fractions was isolated using a PARIS kit (Thermo Fisher Scientific, Waltham, MA). Genomic DNA was extracted from cells using the DNA Isolation Mini Kit (Vazyme Biotech Co., Ltd, Nanjing, China).

### Quantitative real-time PCR (qRT-PCR)

Complementary DNAs (cDNAs) were synthesized with random primers or miRNA-specific primers using a Prime-Script RT Reagent Kit (Takara, Dalian, China) from 1 μg of total RNA. The qRT-PCR was conducted in triplicate using TB Green Premix Ex Taq (Takara, Dalian, China) on a Roche Applied Science LightCycler 480II Real-time PCR system (Roche Applied Science, Indiana, USA). The β-actin was used as an internal reference for circRNA and mRNA, and U6 was used for miRNA. Specific primers for detecting circRNA, miRNA, and mRNA were synthesized by TsingKe (Nanjing, China) and are shown in Supplementary Table 4. The Bulge-loop^TM^ miRNA qRT-PCR Primer Sets (one RT primer and one pair of qPCR primers for each set) specific for U6 were designed by RiboBio (Guangzhou, China). The relative RNA abundance was analyzed using the 2^−ΔΔCt^ method.

### Sanger sequencing, RNase R digestion, and actinomycin D assays

RNA extracted from KYSE-150 cells was subjected to Sanger sequencing and RNase R digestion. The PCR products amplified by divergent primers for circFAM120B were subjected to Sanger sequencing analysis. For RNase R digestion, the total RNA (2.5 μg) was incubated at 37 °C for 15 min with or without 4 U/μg RNase R (Epicentre Technologies, Madison, WI, USA). RNA was purified using the RNeasy MinElute Cleanup kit (Qiagen, Hilden, Germany) and then analyzed by qRT-PCR or observed by 2% agarose gel electrophoresis. For actinomycin D (ActD) treatment, KYSE-150 cells were incubated with 5 μg/ml ActD for 0, 6, 12, 18, and 24 h, and then the RNAs were analyzed by qRT-PCR.

### siRNA and lentivirus production and transfection

Small interfering RNA (siRNA) oligonucleotides targeting circFAM120B and PKR or negative controls were acquired from RiboBio (Guangzhou, China), and miR-661 mimics and inhibitors were purchased from TsingKe (Nanjing, China). Transfections of siRNAs were performed using Lipofectamine 3000 Transfection Reagent (Invitrogen, CA, USA), and the efficiency was verified by qRT-PCR analysis. The sequences targeted by siRNAs, mimics and inhibitors are summarized in Supplementary Table 4.

For stable transfections, the overexpression lentivirus for circFAM120B containing the green fluorescent protein gene was provided by GeneChem (Shanghai, China), and the infection was performed in accordance with the manufacturers’ protocol. Approximately 36 h after infection, cells were treated with puromycin (5 μg/ml) for 10 d to select stably transfected cells. Surviving cells were observed under a fluorescence microscope, and circFAM120B overexpression was confirmed by qRT-PCR.

### Cell counting kit-8 (CCK-8) assay

For the CCK-8 assay, the transfected cells (2 × 10^3^ cells/well) were seeded into 96-well plates in quintuplicate and cultured until entirely adherent. CCK-8 solution (10 μl, Dojindo, Tokyo, Japan) was added to each well at 0, 24, 48, 72, and 96 h. After 2 h of incubation, the absorbance was measured on a microplate reader at 450 nm in triplicate (BioTek, Vermont, USA).

### Colony formation assay

Transfected cells (1 × 10^3^ cells/well) were seeded and incubated in 6-well plates for colony formation assays. After 10 d, the cells were fixed with 4% paraformaldehyde (Sigma, Missouri, USA) and stained with crystal violet (Beyotime, Nanjing, Jiangsu, China) for 20 min. The number of colonies with more than 50 cells was manually counted under a microscope, and the colony-forming efficiency of cells in each plate was calculated.

### 5-Ethynyl-2ʹ-deoxyuridine (EdU) assay

We used the BeyoClick™ EdU Cell Proliferation Kit (Beyotime, Jiangsu, China) to perform the EdU assay. In brief, the transfected cells were seeded in 96-well plates and incubated with 100 μl medium supplemented with 10 μM EdU. After incubation (approximately 2 h for KYSE-150 and 3.5 h for TE-1), cells were fixed with 4% paraformaldehyde for 30 min, permeabilized with 0.5% Triton-X-100 in PBS for 20 min, and then washed with 3% BSA in PBS. Afterward, the cells were incubated in Click Additive Solution and stained with Hoechst. Images were captured with a fluorescence microscope and analyzed by the ImageJ system.

### Cell migration and invasion assays

Briefly, 24-well plate inserts with an 8-μm pore size (Corning Costar, New York, USA; Millipore, MA, USA) were coated with Matrigel (diluted 1:8 with serum-free medium, 50 μL/well, incubated at 37 °C for 30 min to form a gel, for the invasion assay) or left uncoated (for the migration assay). The transfected ESCC cells resuspended in 200 μL of serum-free medium (approximately 8 × 10^4^ cells for the migration assay and 16 × 10^4^ cells for the invasion assay) were added to the upper Transwell chamber. A culture medium containing 10% FBS was added to the lower chamber as a chemoattractant. After incubation at 37 °C for 24 h for TE-1 cells and 30 h for KYSE-150 cells, the medium containing ESCC cells in the upper chamber was discarded. The lower surface of the membrane was fixed with 100% methanol for 20 min and stained with 0.5% crystal violet for 20 min. The number of invasive or migrative cells was then quantified with the aid of a microscope (Nikon, Japan).

### RNA fluorescence in situ hybridization (FISH)

A Cy3-labeled probe for circFAM120B was designed by RiboBio (Guangzhou, China). Experiments were conducted using fluorescent in situ hybridization kit (RiboBio) based on the manufacturer’s manual with minor modifications. Briefly, cells were fixed with 4% paraformaldehyde at 4 °C for 2 h, permeabilized with 0.5% Triton X-100 at 4 °C for 5 min, prehybridized at 37 °C for 30 min, and then hybridized at a probe concentration of 5 μM overnight. DAPI and 18S probes were used as the nuclear and cytoplasmic references, respectively. Images were captured using confocal microscopy (Zeiss, Oberkochen, Germany).

### RNA pull-down, silver staining, and mass spectrometry analysis

Biotinylated circFAM120B was generated by using a MEGAscript™ T7 Transcription Kit (Invitrogen) and a Pierce RNA 3’ End Desthiobiotinylation Kit (Thermo Fisher Scientific, MA, USA) following the manufacturer’s instructions. Then, RNA-protein pull-down was performed with a Pierce Magnetic RNA-Protein Pull-Down Kit (Thermo Fisher Scientific, Waltham, MA, USA). Briefly, the biotinylated RNAs were captured with streptavidin-coated magnetic beads and incubated with whole-cell lysates from cells at 4 °C for 6 h. The RNA-protein complex was then washed and eluted. The retrieved eluate was separated on a sodium dodecyl sulfate (SDS)-polyacrylamide gel, followed by silver staining with a Rapid Silver Staining Kit (Beyotime, Shanghai, China), and subjected to mass spectrometry analysis at Shanghai Bioprofile Technology Company Ltd. (Shanghai, China). The protein with >2 unique peptides was considered a candidate.

### Preparation of cell lysates and western blot

Cells were lysed in RIPA buffer supplemented with protease and phosphatase inhibitors for 10 min on ice, and protein concentrations were determined using the Bicinchoninic Acid Protein Assay Kit (Thermo Fisher Scientific). Equal amounts of protein lysates were separated on SDS-PAGE gels and then transferred onto polyvinylidene fluoride membranes using the wet transfer method (Millipore, Massachusetts, USA). After overnight incubation with the primary antibody at 4 °C in a sealed bag, the membranes were subsequently incubated with appropriate secondary antibodies at room temperature for 2 h. The protein bands were visualized with ECL chemiluminescent reagent (Tanon, Shanghai, China). The primary antibodies used in the current study were as follows: PPM1L (Affinity, Changzhou, China, DF4349), Bcl2 (Abcam, USA, ab32124), Caspase3 (Abcam, USA, ab32351), p-JNK (Abcam, USA, ab124956), JNK (Protech, Wuhan, China, 66210-1-Ig), CCND1 (Protech, Wuhan, China, 26939-1-AP), PKR (Abcam, USA, ab32052), P38 (Affinity, Changzhou, China, AF6456), p-P38 (Affinity, Changzhou, China, AF4001), E-cadherin (Abcam, USA, ab40772), N-cadherin (Abcam, USA, ab76011), Vimentin (Abcam, USA, ab92547), and β-actin (Protech, Wuhan, China, 20536-1-AP).

### Dual-luciferase reporter assay

Wild-type or mutant circFAM120B (WT/MUT-circFAM120B) and PPM1L 3′-UTR (WT/MUT-PPM1L 3′-UTR) fragments containing putative binding sites of miR-661 were cloned downstream of the firefly luciferase open reading frame in the PGL3-promoter plasmid (Promega, Madison, WI, USA) and verified by RNA-seq analysis (TsingKe, Nanjing, China). HEK293T cells were seeded in 24-well plates and co-transfected with the corresponding WT/MUT plasmid and either miR-661 mimics or negative control using Lipofectamine 2000 Transfection Reagent (Invitrogen, CA, USA). Twenty-four hours after co-transfection, luciferase reporter assays were performed using a dual-luciferase reporter assay system (Promega) according to the manufacturer’s instructions. Relative luciferase activity was normalized to Renilla luciferase activity.

### RNA immunoprecipitation (RIP) assay

The RIP assay was carried out with a Magna RIP RNA-Binding Protein Immunoprecipitation Kit (Millipore, Billerica, MA, USA). In brief, KYSE-150 cells were lysed in RIP lysis buffer on ice for 30 min. Magnetic beads were preincubated with either anti-AGO2 or IgG antibodies for 30 min at room temperature. After centrifugation, the supernatant was immunoprecipitated with beads conjugated to specific antibodies at 4 °C overnight. Then, the immunoprecipitated RNA was purified and processed for qRT-PCR analysis.

### Co-immunoprecipitation (Co-IP) assay

The Co-IP assay was performed using a Pierce^TM^ Co-Immunoprecipitation Kit (Thermo, USA). The ubiquitin antibody used for the Co-IP assay was purchased from Proteintech, China.

### Xenografts in nude mice

All animal procedures were conducted in accordance with the National Institutes of Health Guidelines for the Care and Use of Laboratory Animals and approved by the Animal Care Committee of Nanjing Medical University (approval numbers: 2010022 and 2006011). All male BALB/c nude mice (4 weeks old) were purchased from Shanghai SLAC Laboratory Animal Co. Ltd. (Shanghai, China). The animals were randomly allocated to experimental groups, and researchers were blinded to the group assignments of animals during experiments.

For xenograft tumor formation, stably transfected KYSE-150 cells (1 × 10^7^ cells/200 μl PBS) carrying OE-circFAM120B (circFAM120B overexpression) or mock vector were subcutaneously injected into the flanks of mice in the armpit (*n* = 6 per group). Tumor growth was measured every 5 days with a Vernier caliper starting one week after injection, and tumor volumes were calculated by the following formula: volume = 1/2 (length × width^2^). Thirty days later, the mice were euthanized, and the tumors were harvested en bloc and examined. For the in vivo metastasis assay, transfected TE-1 cells (2 × 10^6^ cells/100 μl PBS) stably expressing OE-circFAM120B or mock vector were injected from the tail vein of nude mice (*n* = 5 per group). After 2 months, all mice were euthanized, and their lungs were surgically dissected. The harvested lungs were embedded in paraffin for hematoxylin and eosin (HE) staining or immunohistochemistry staining.

### Statistical analyses

All experiments were independently repeated at least three times, and data are expressed as the mean ± standard deviation (SD) of triplicate. The statistical significance of differences was calculated by a two-tailed Student’s *t*-test or Mann–Whitney *U* test wherever appropriate. Survival curves were plotted using the Kaplan–Meier method and compared by the log-rank test. The correlation between circFAM120B and PPM1L expression in ESCC tissues was assessed by Pearson correlation. All statistical analyses were performed using GraphPad Prism (version 6.0) or R software version 3.6.3 (https://www.r-project.org/), and *P* values < 0.05 were considered statistically significant.

## Supplementary information

Supplementary Tables

Supplementary figure legends

Supplementary figure 1. The abundance of RNAs in ESCC cells after modification.

Supplementary figure 2. circFAM120B acts as a tumor suppressor in ESCC cells.

Supplementary figure 3. The expression on PKR (also named EIF2AK2) in esophageal carcinoma from the TCGA database.

Supplementary figure 4. circFAM120B regulates the PKR/P38 MAPK/EMT pathway.

Supplementary figure 5. Original western blots.

Academic Journals Reporting Checklist

## Data Availability

The original contributions presented in the study are included in the article/Supplementary Material. Further inquiries can be directed to the corresponding author. Original western blots are provided in Supplementary Fig. 5.
