# Supplementary material for: circFAM120B functions as a tumor suppressor in esophageal squamous cell carcinoma via the miR-661/PPM1L axis and the PKR/p38 MAPK/EMT pathway
Source: Cell Death Dis. 2022 Apr 18;13(4):361. doi: 10.1038/s41419-022-04818-5 (PMC9016076; doi:10.1038/s41419-022-04818-5)
Supplement: Supplementary file 1 — Supplementary Tables [file 41419_2022_4818_MOESM1_ESM.docx]

**Supplementary Tables**

**Supplemental Table 1. Top downregulated circRNAs in ESCC.**

| **circRNA** | **log_2_FC** | ***FDR*** | **Location** | **Gene symbol** |
| --- | --- | --- | --- | --- |
| hsa_circ_0000550 | -2.71 | <0.01 | chr14:70245970-70246161 | SLC10A1 |
| hsa_circ_0049613 | -2.54 | <0.01 | chr19:12962747-12963209 | MAST1 |
| hsa_circ_0041551 | -2.43 | <0.01 | chr17:4145550-4145743 | ANKFY1 |
| hsa_circ_0084789 | -2.34 | <0.01 | chr8:77616277-77620283 | ZFHX4 |
| hsa_circ_0005654 | -1.71 | <0.01 | chr4:121675707-121732604 | PRDM5 |
| hsa_circ_0001666 | -1.68 | <0.05 | chr6:170626457-170639638 | FAM120B |
| hsa_circ_0008078 | -1.63 | <0.01 | chr21:37619814-37633030 | DOPEY2 |
| hsa_circ_0088681 | -1.49 | <0.05 | chr9:130285957-130294057 | FAM129B |
| hsa_circ_0065871 | -1.36 | <0.05 | chr3:50677796-50679763 | MAPKAPK3 |
| hsa_circ_0021506 | -1.36 | <0.05 | chr11:22225349-22249132 | ANO5 |

**Supplemental Table 2. Relationship between circFAM120B expression and clinicopathologic characteristics of patients with ESCC.**

| **Parameter** | **No. of patients** | **circFAM120B (low expressed)** | **circFAM120B (high expressed)** | ***P*-valve** |
| --- | --- | --- | --- | --- |
| Sex |  |  |  |  |
| Male | 83 | 63 | 20 | 0.654 |
| Female | 47 | 34 | 13 |  |
| Age |  |  |  |  |
| <65 | 45 | 37 | 8 | 0.147 |
| ≥65 | 85 | 60 | 25 |  |
| Smoking |  |  |  |  |
| Yes | 65 | 49 | 16 | 0.840 |
| No | 65 | 48 | 17 |  |
| Drinking |  |  |  |  |
| Yes | 57 | 47 | 10 | <0.001***** |
| No | 73 | 20 | 23 |  |
| Tumor size |  |  |  |  |
| <4 | 98 | 67 | 31 | 0.004***** |
| ≥4 | 32 | 30 | 2 |  |
| Tumor location |  |  |  |  |
| Upper/upper-middle | 20 | 16 | 4 | 0.833 |
| Middle | 64 | 47 | 17 |  |
| Lower-middle/lower | 46 | 34 | 12 |  |
| Differentiation grade |  |  |  |  |
| Well | 51 | 36 | 15 | 0.256 |
| Moderate | 51 | 42 | 9 |  |
| Poorly | 28 | 19 | 9 |  |
| T stage |  |  |  |  |
| T1-2 | 71 | 54 | 17 | 0.679 |
| T3 | 59 | 43 | 16 |  |
| N stage |  |  |  |  |
| N0 | 77 | 58 | 19 |  |
| N1-3 | 53 | 39 | 14 |  |
| TNM stage |  |  |  |  |
| Ⅰ-Ⅱ | 87 | 67 | 20 | 0.372 |
| Ⅲ-Ⅳ | 43 | 30 | 13 |  |

**P*<0.05

**Supplemental Table 3. Characteristics of patients with ESCC collected from Yixing.**

| **No.** | **Sex** | **Age (years)** | **Tumor size (mm)** | **Location** | **Differentiation** | **Clinical stage** |
| --- | --- | --- | --- | --- | --- | --- |
| 1 | Male | 58 | >4 | Middle | Moderate | IIB |
| 2 | Male | 72 | <4 | Middle | Well | IB |
| 3 | Male | 74 | >4 | Lower-middle | Moderate | IIIA |
| 4 | Male | 62 | >4 | Upper-middle | Moderate | IIA |
| 5 | Female | 55 | <4 | Upper | Poorly | IB |
| 6 | Female | 74 | <4 | Middle | Poorly | IIIB |
| 7 | Female | 62 | <4 | Middle | Moderate | IIA |
| 8 | Female | 65 | >4 | Upper | Well | IIB |

**Supplemental Table 4. The primer sequences used in the research.**

| **Name** | **Primer sequences (5’-3’)** |
| --- | --- |
| Q-circFAM120B forward | TGCAGATGACCATTCCAGATCC |
| Q-circFAM120B reversed | TGGTGCTCTGCCAGTTCTTTGA |
| Q-FAM120B forward | AGGACGGGCTCTGGGTAT |
| Q-FAM120B reversed | CCACTGGTCATGCTCATACTGT |
| Q-PKR forward | GGCTGTTGGGATGGATTT |
| Q-PKR reversed | GGCACTTAGTCTTTGACCTT |
| Q-PPM1L forward | GTGTGACAAAGATGGGAACGC |
| Q-PPM1L reversed | CAGGGACCGAGACATGGC |
| Q-hsa-miR-661-RT | GTCGTATCGACTGCAGGGTCCGAGGTATTCGCAGTCGATACGACACGCGC |
| Q-hsa-miR-661 forward | TGCCTGGGTCTCTGGCCT |
| Q-hsa-miR-661 reversed | ACTGCAGGGTCCGAGGTATT |
| Q-β-actin forward | AGATGTGATCAGCAAGCAG |
| Q-β-actin reversed | GCGCAAGTTAGGTTTTGTCA |
| si-circFAM120B siRNA-1 | GATGACCATTCCAGATCCT |
| si-circFAM120B siRNA-2 | TGACCATTCCAGATCCTTT |
| si-circFAM120B siRNA-3 | TTCCAGATCCTTTCCCGGA |
| mimic hsa-miR-661 negative control forward | UCACAACCUCCUAGAAAGAGUAGA |
| mimic hsa-miR-661 negative control reversed | UCUACUCUUUCUAGGAGGUUGUGA |
| mimic hsa-miR-661 forward | UGCCUGGGUCUCUGGCCUGCGCGU |
| mimic hsa-miR-661 reversed | ACGCGCAGGCCAGAGACCCAGGCA |
| inhibitor hsa-miR-661negative control | UCUACUCUUUCUAGGAGGUUGUGA |
| inhibitor hsa-miR-661 | ACGCGCAGGCCAGAGACCCAGGCA |
| mutant-hsa-miR-661 | UAUUCAAAUCUCUGAGGCGCGCGU |
| PPM1L 3’-UTR forward | GCTCTAGA TTCCTTGAAGCCAGGTGCA |
| PPM1L 3’-UTR reversed | GCTCTAGA AGGCAAAGAGAGTGACCCAG |
| si-PPM1L siRNA-1 | GAAGCAGTTCGATTCATCA |
| si-PPM1L siRNA-2 | GCATAGTTTTACAGTCATT |
| si-PPM1L siRNA-3 | GCTTGGCTCTATGGAGTTA |
| circFAM120B sense forward | TAATACGACTCACTATAGGGATCCTTTCCCGGAGTTCAGTTA |
| circFAM120B sense reversed | CTGGAATGGTCATCTGCAGC |
| circFAM120B anti-sense forward | ATCCTTTCCCGGAGTTCAGTTA |
| circFAM120B anti-sense reversed | TAATACGACTCACTATAGGGCTGGAATGGTCATCTGCAGC |
