# Supplementary material for: circFAM120B functions as a tumor suppressor in esophageal squamous cell carcinoma via the miR-661/PPM1L axis and the PKR/p38 MAPK/EMT pathway
Source: Cell Death Dis. 2022 Apr 18;13(4):361. doi: 10.1038/s41419-022-04818-5 (PMC9016076; doi:10.1038/s41419-022-04818-5)
Supplement: Supplementary file 2 — Supplementary figure legends [file 41419_2022_4818_MOESM2_ESM.docx]

**Supplementary figure legends**

**Supplementary figure 1. The abundance of RNAs in ESCC cells after modification.**

(A) The abundance of circFAM120B in ESCC cells with circFAM120B overexpression and knockdown. (B) The abundance of FAM120B in ESCC cells with circFAM120B overexpression and knockdown. (C) The abundance of PPM1L in ESCC cells with PPM1L overexpression and knockdown. (D) The abundance of PKR in ESCC cells with circFAM120B overexpression and knockdown. ***P*<0.01, ****P*<0.001, and ns: no significance.

**Supplementary figure 2. circFAM120B acts as a tumor suppressor in ESCC cells.**

(A) Proliferation abilities of ESCC cells with PPM1L overexpression and knockdown were evaluated by CCK-8 assay (n=5 biologically independent replicates). (B) Colony formation assays were used for ESCC cells with PPM1L overexpression and knockdown (n=3 biologically independent replicates). (C) Detection of the cell proliferation of ESCC cells with PPM1L overexpression and knockdown by EDU assay (n=3 biologically independent replicates). (D) Migration and invasion assays for ESCC cells with PPM1L overexpression and knockdown were determined by Transwell assays (n=3 biologically independent replicates). ***P*<0.01, ****P*<0.001.

**Supplementary figure 3. The expression on PKR (also named EIF2AK2) in esophageal carcinoma from the TCGA database.**

**Supplementary figure 4. circFAM120B regulates the PKR/P38 MAPK/EMT pathway.**

(A) Western bolt analyses validate the efficiency of PKR overexpression and inhibitory. (B&C) Protein levels were evaluated by western blot in ESCC cells with the indicated treatments.

**Supplementary figure 5. Original western blots.**
