# Supplementary figures and images for: circFAM120B functions as a tumor suppressor in esophageal squamous cell carcinoma via the miR-661/PPM1L axis and the PKR/p38 MAPK/EMT pathway
Source: Cell Death Dis. 2022 Apr 18;13(4):361. doi: 10.1038/s41419-022-04818-5 (PMC9016076; doi:10.1038/s41419-022-04818-5)

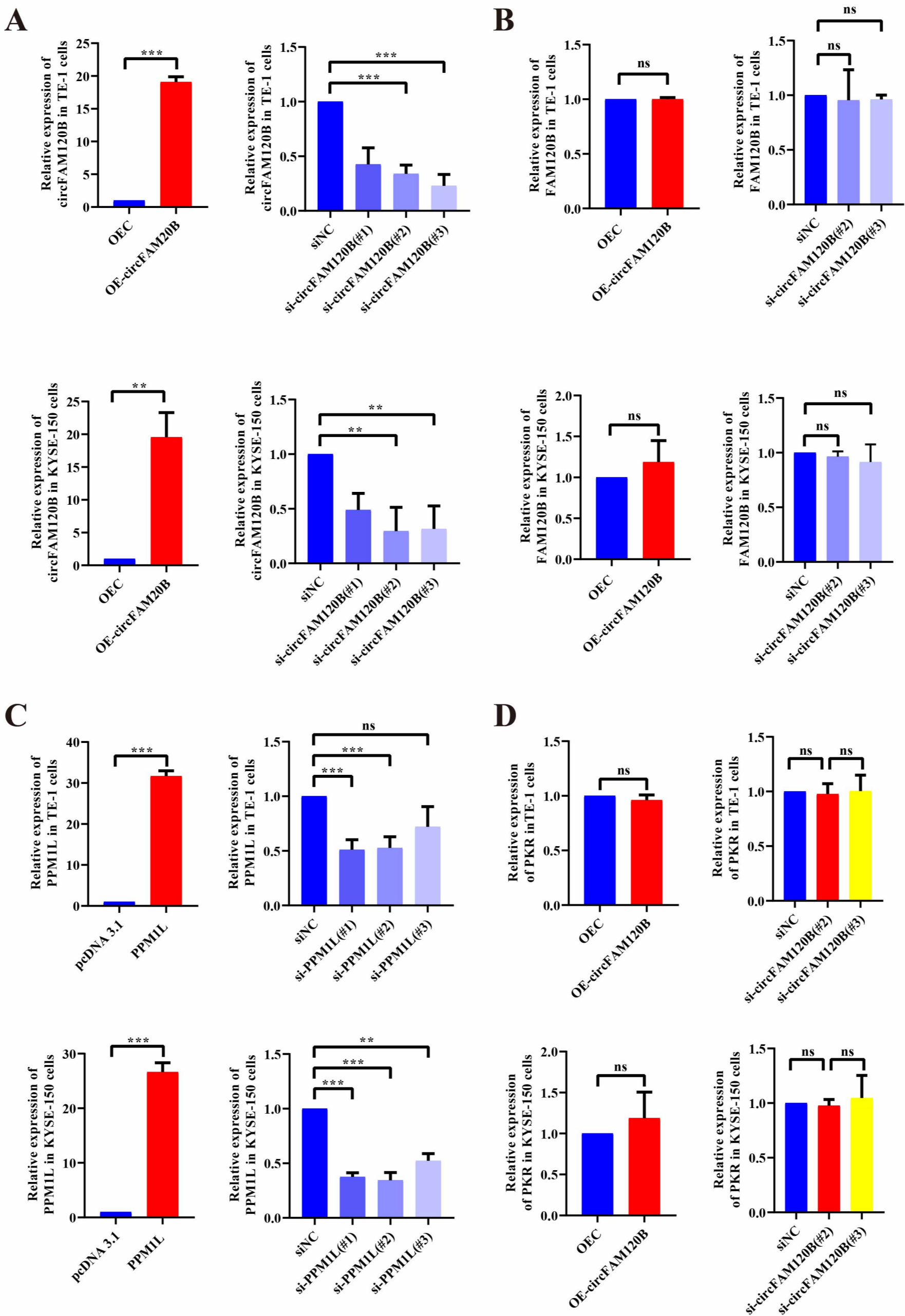

Supplement: Supplementary file 3 — Supplementary figure 1. The abundance of RNAs in ESCC cells after modification. [file 41419_2022_4818_MOESM3_ESM.pdf]

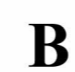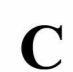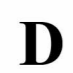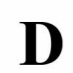

Supplement: Supplementary file 4 — Supplementary figure 2. circFAM120B acts as a tumor suppressor in ESCC cells. [file 41419_2022_4818_MOESM4_ESM.pdf]

A

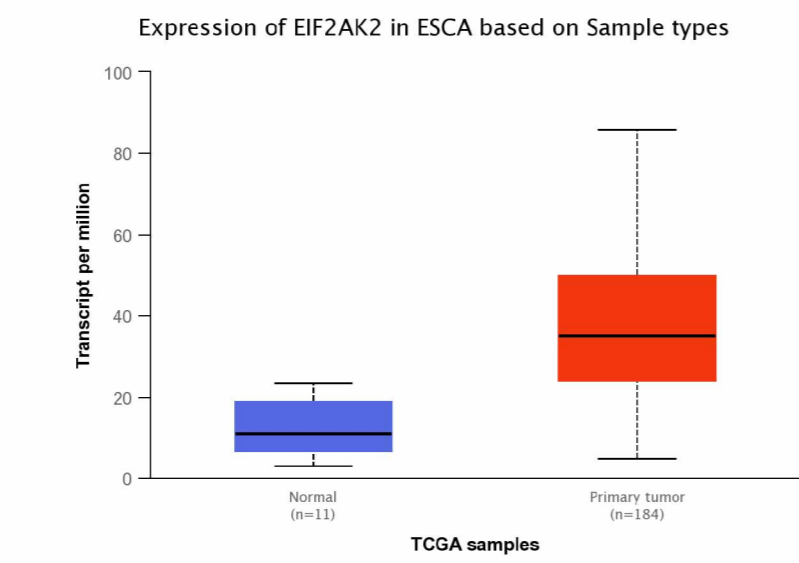

B

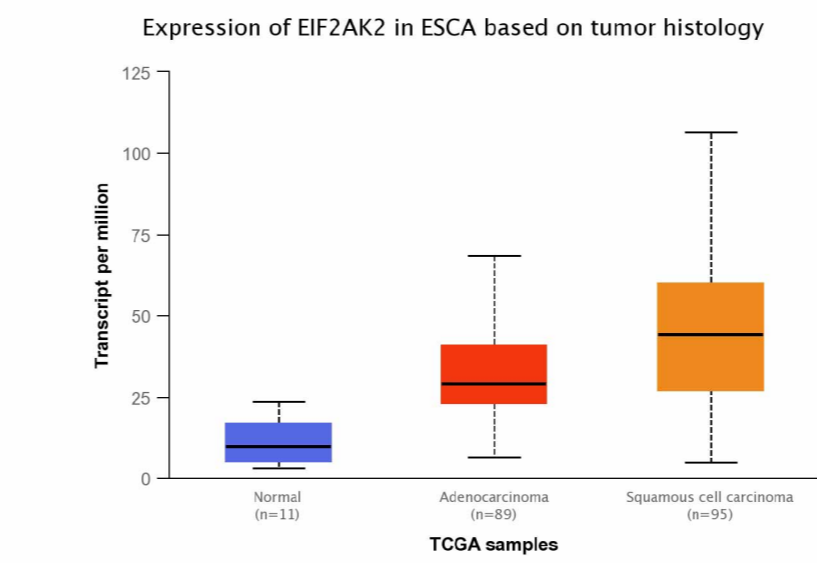

C

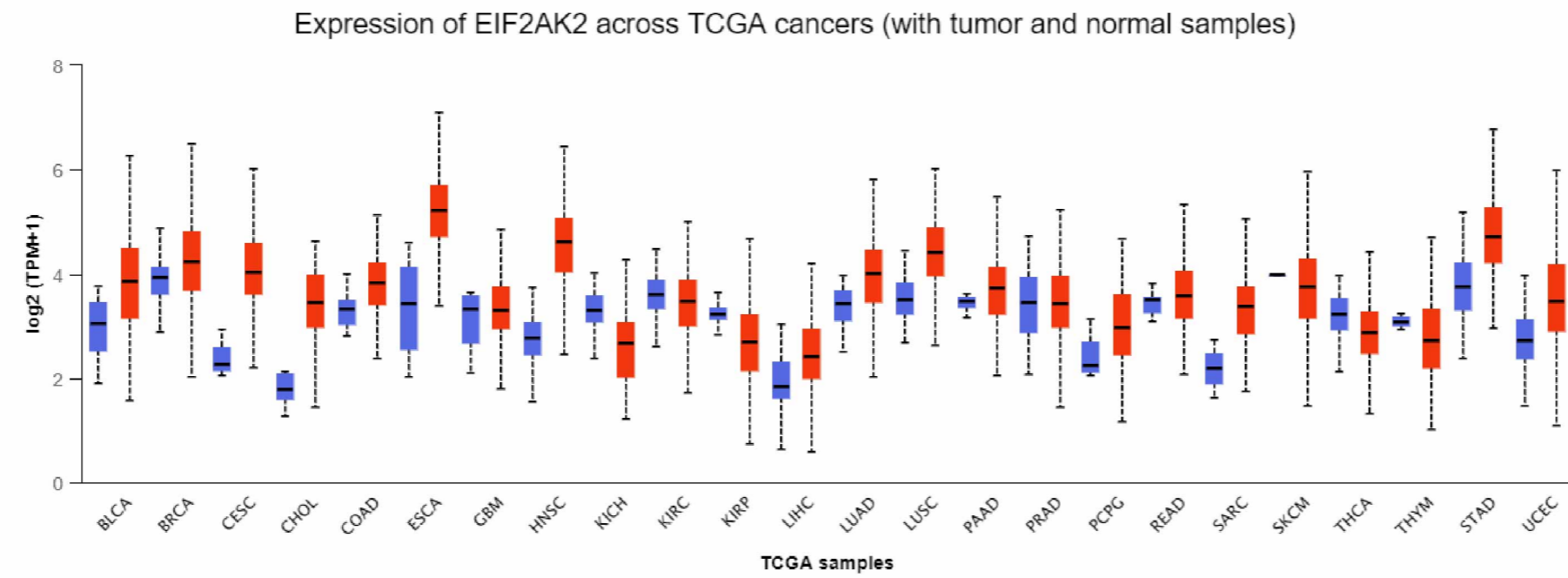

Supplement: Supplementary file 5 — Supplementary figure 3. The expression on PKR (also named EIF2AK2) in esophageal carcinoma from the TCGA database. [file 41419_2022_4818_MOESM5_ESM.pdf]

A

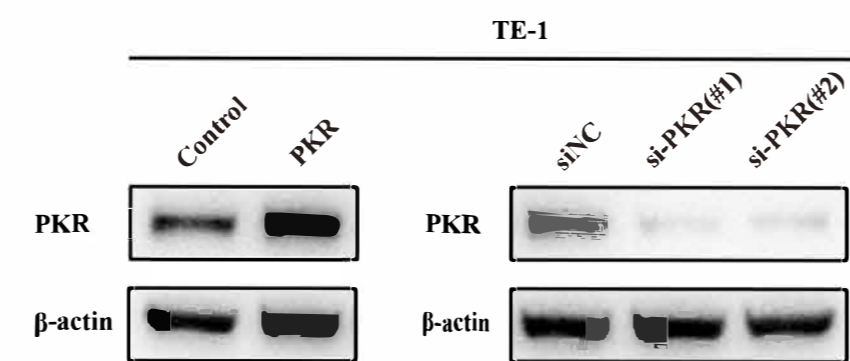

B

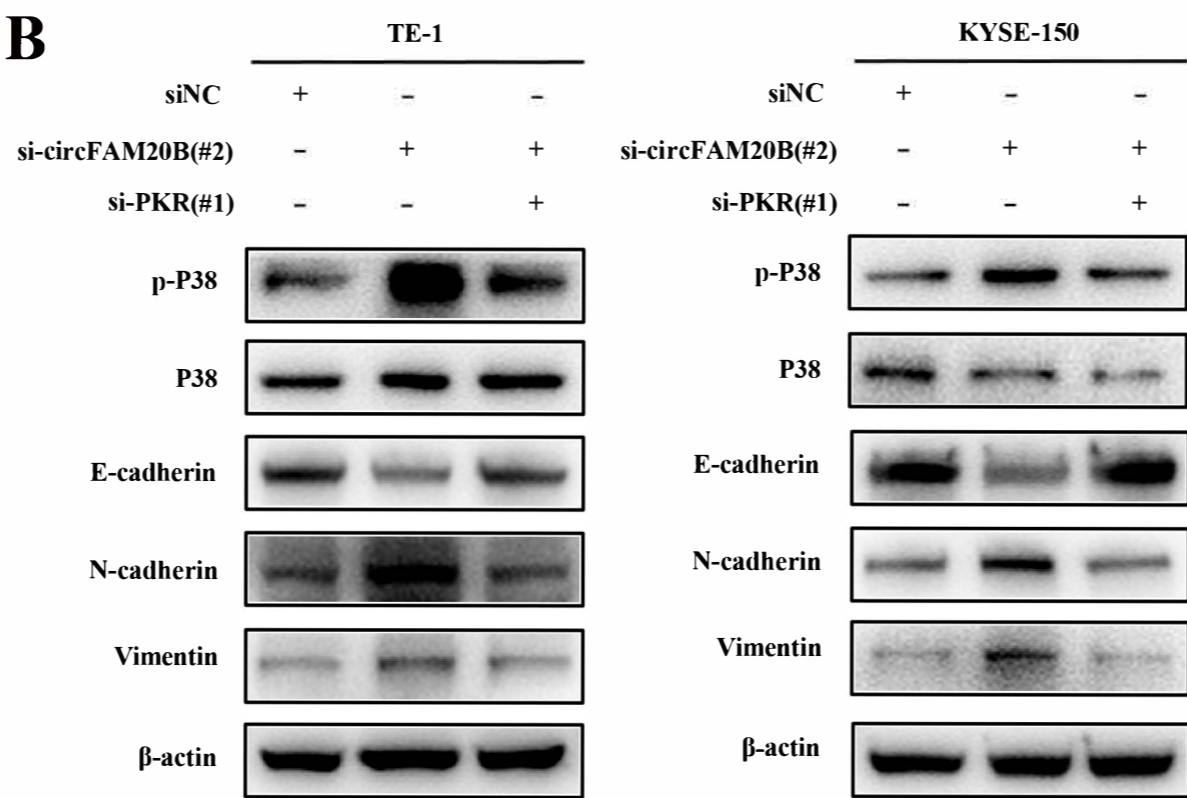

C

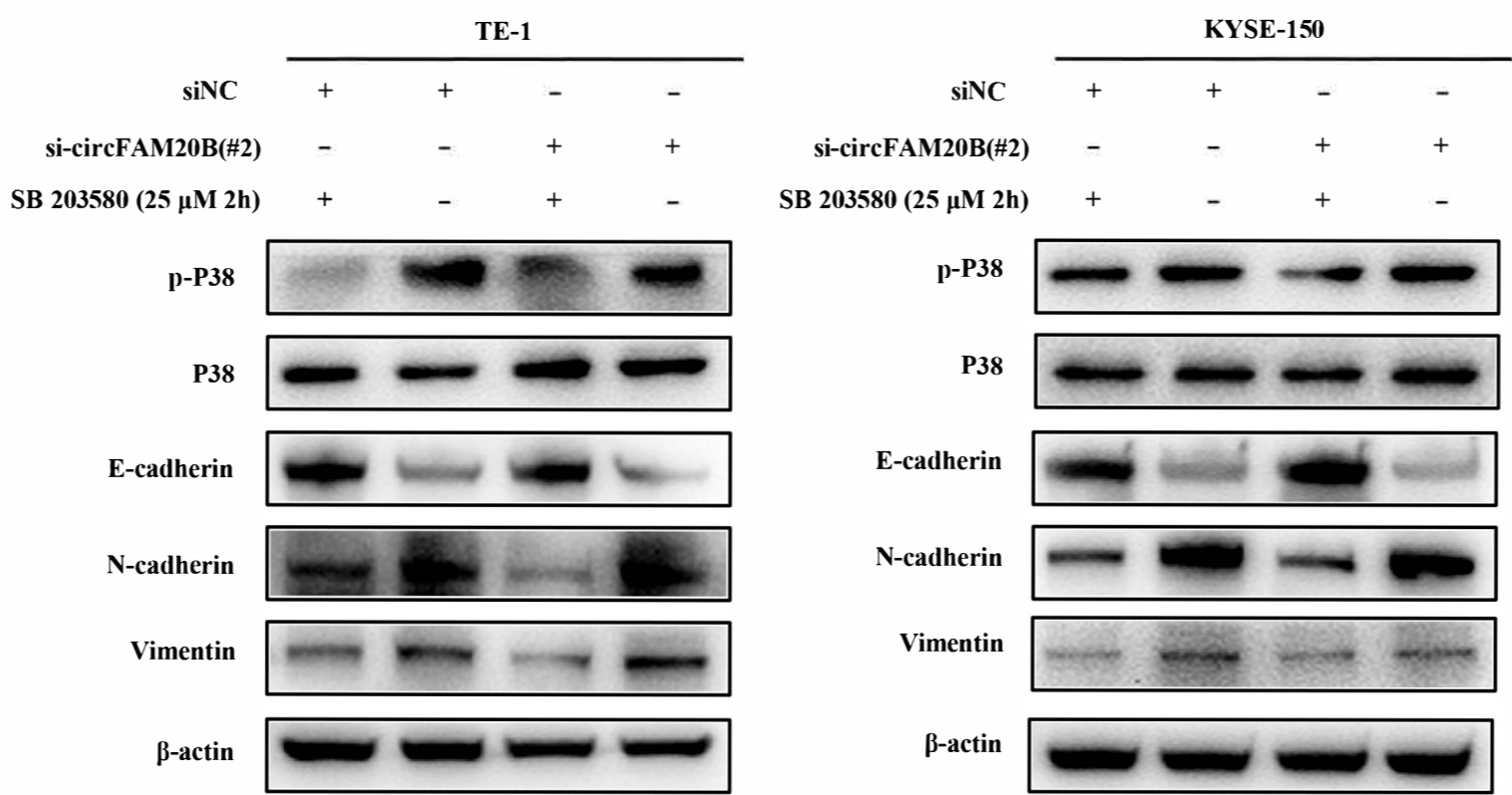

Supplement: Supplementary file 6 — Supplementary figure 4. circFAM120B regulates the PKR/P38 MAPK/EMT pathway. [file 41419_2022_4818_MOESM6_ESM.pdf]
