# Supplementary material for: circFAM120B functions as a tumor suppressor in esophageal squamous cell carcinoma via the miR-661/PPM1L axis and the PKR/p38 MAPK/EMT pathway
Source: Cell Death Dis. 2022 Apr 18;13(4):361. doi: 10.1038/s41419-022-04818-5 (PMC9016076; doi:10.1038/s41419-022-04818-5)

Corresponding to Figure 4F

TE-1

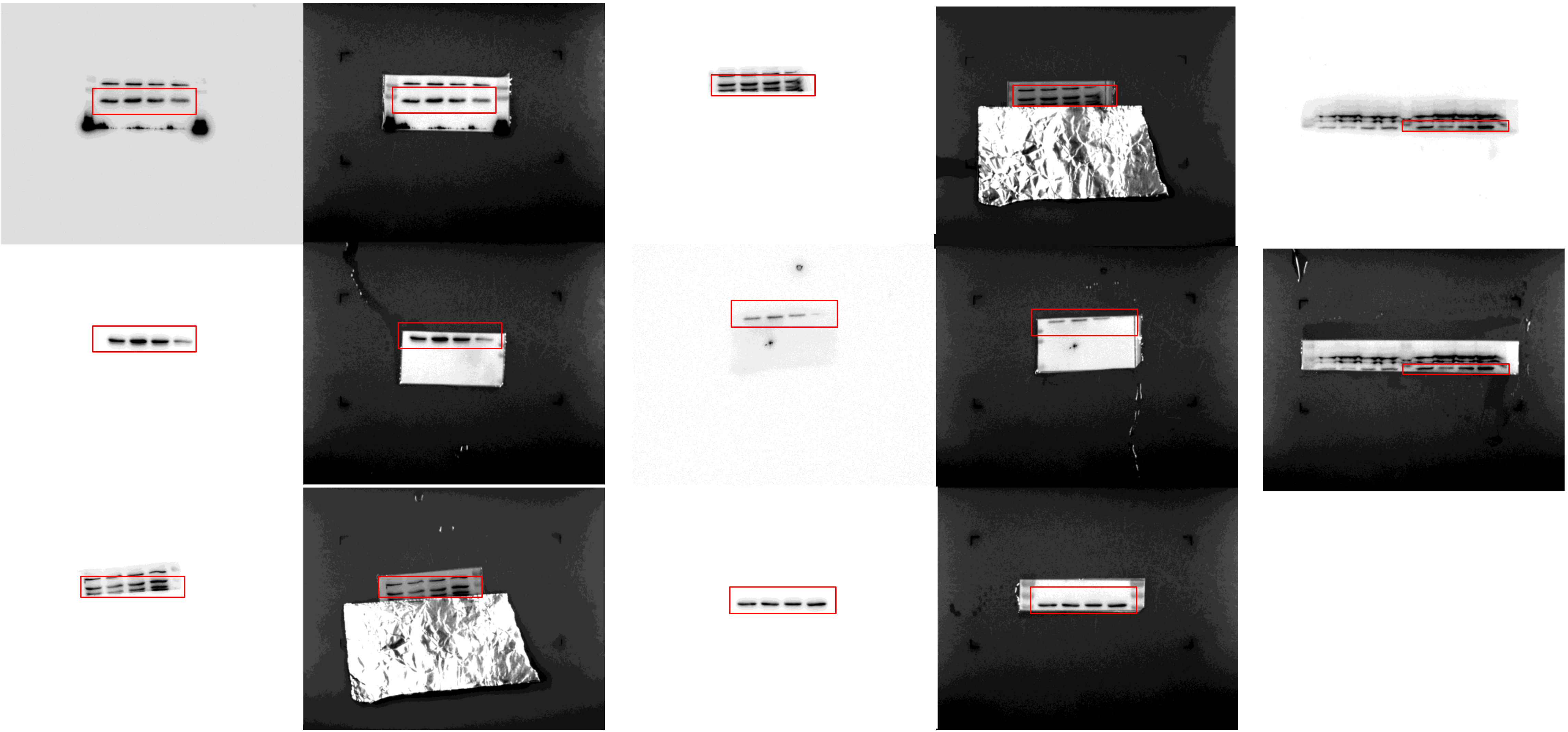

KYSE-150

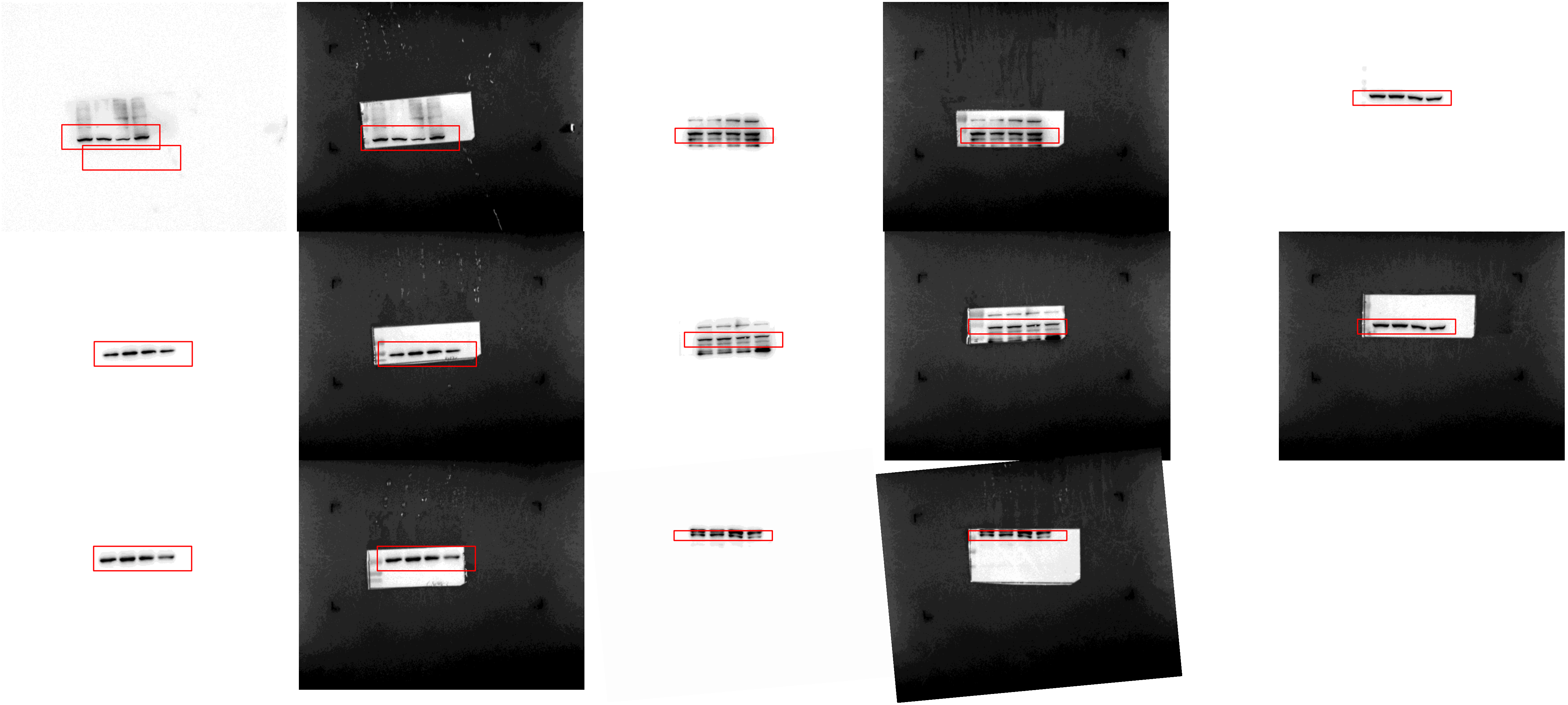

Corresponding to Figure 4G

TE-1

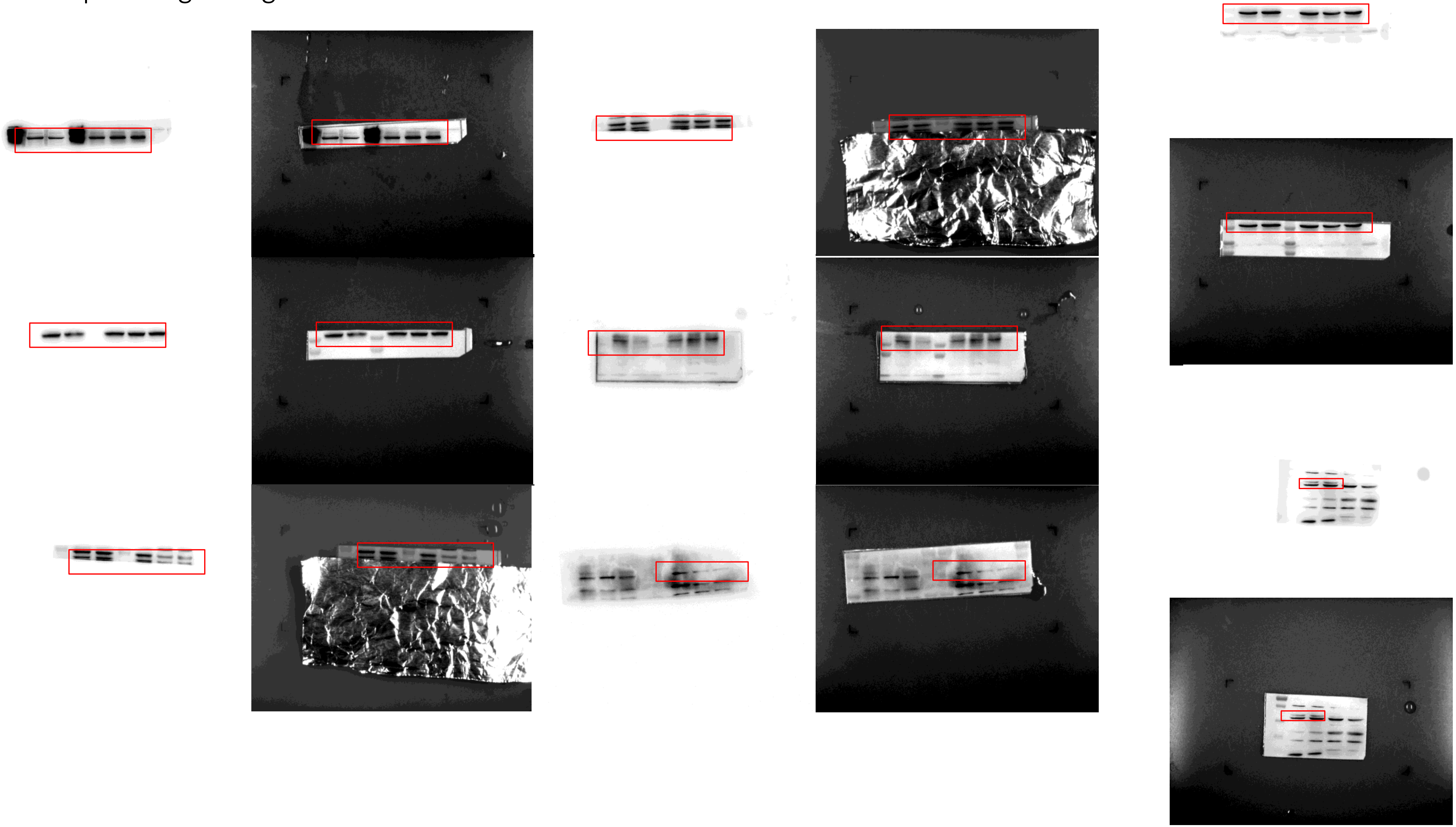

KYSE-150

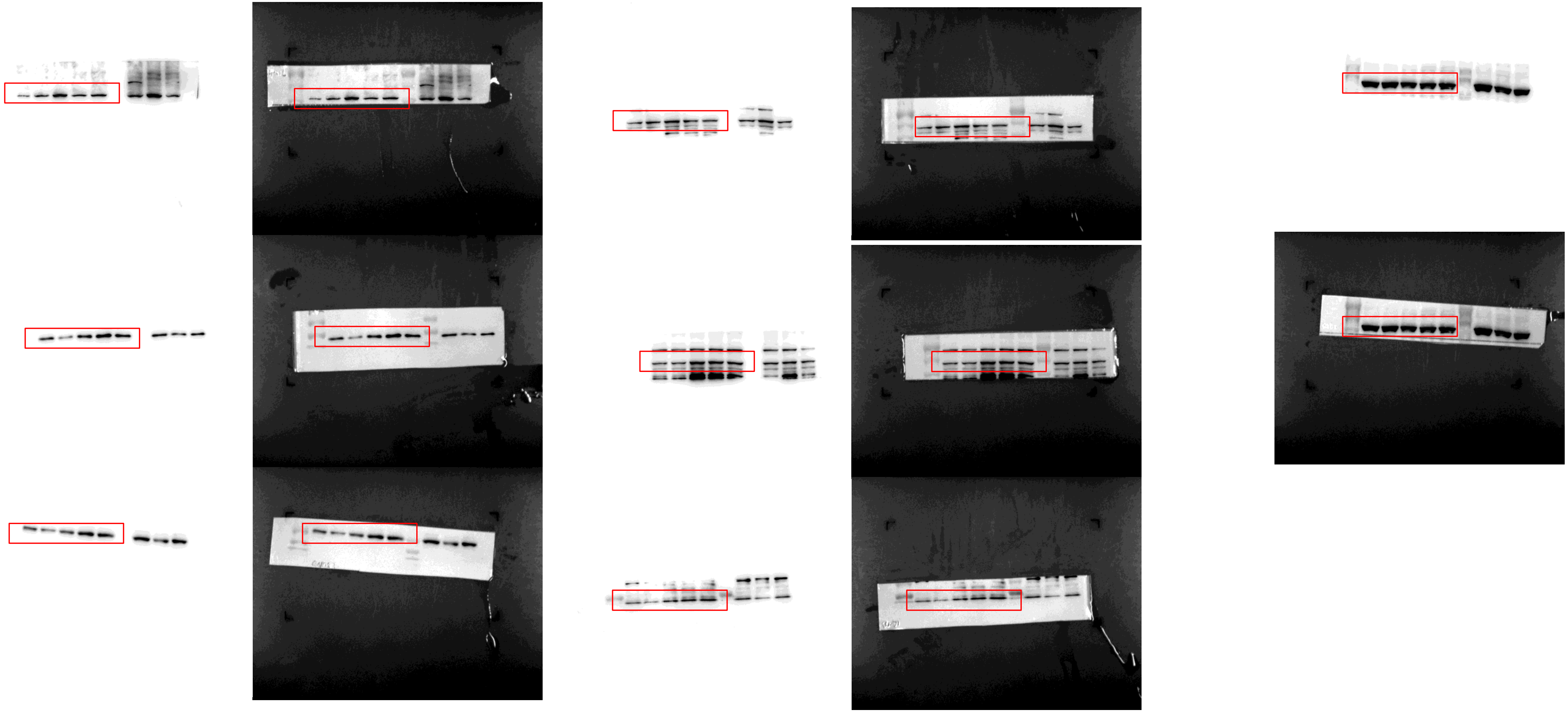

Corresponding to Figure 4H

TE-1

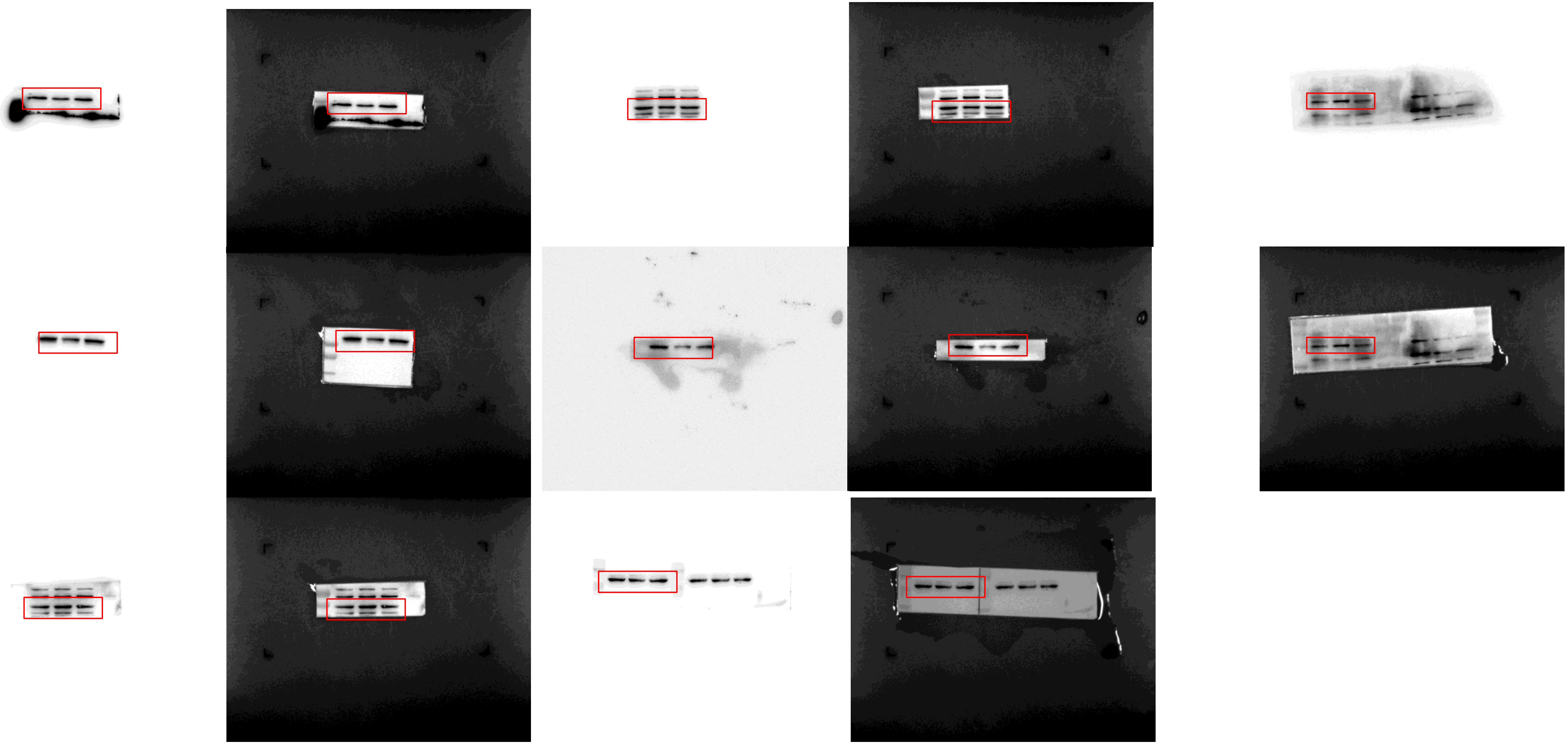

KYSE-150

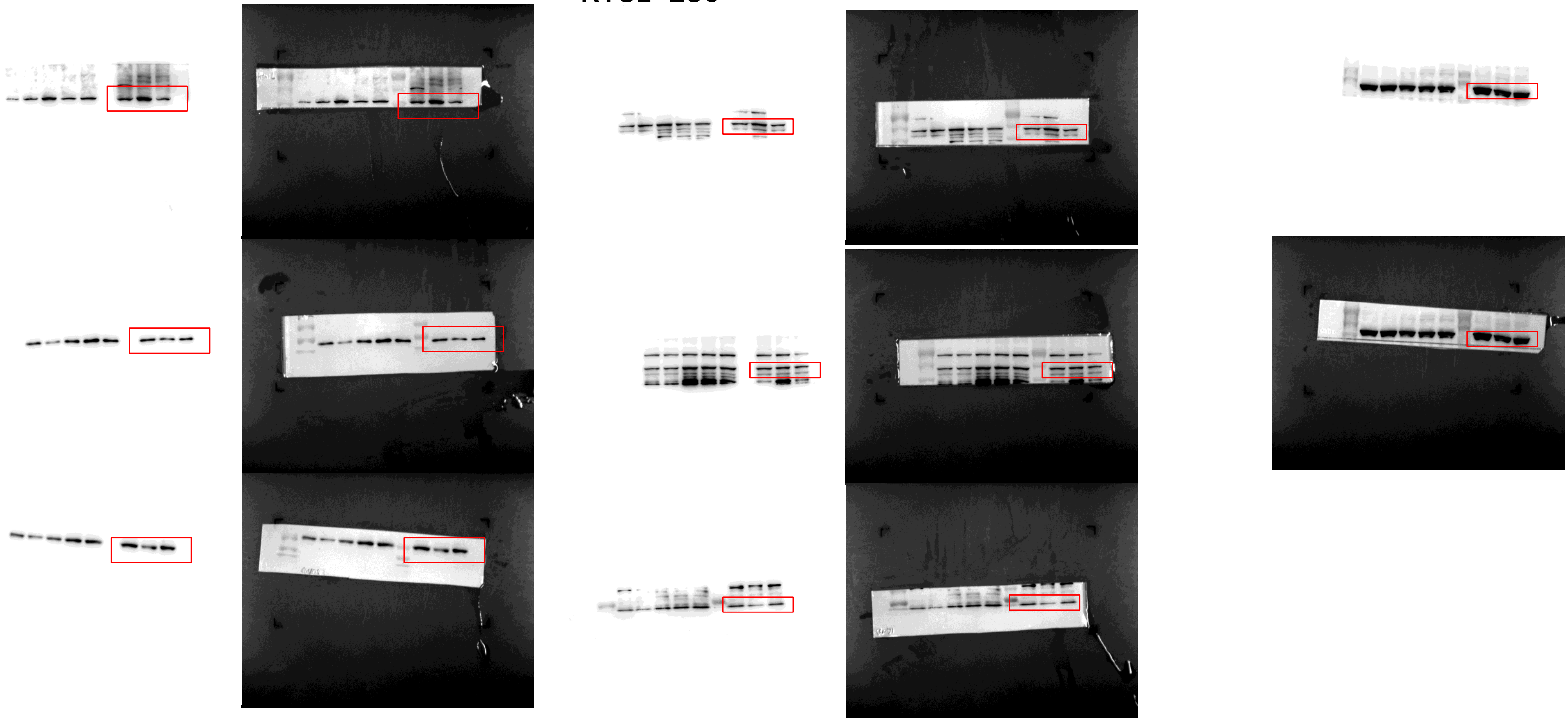

Corresponding to Figure 5B

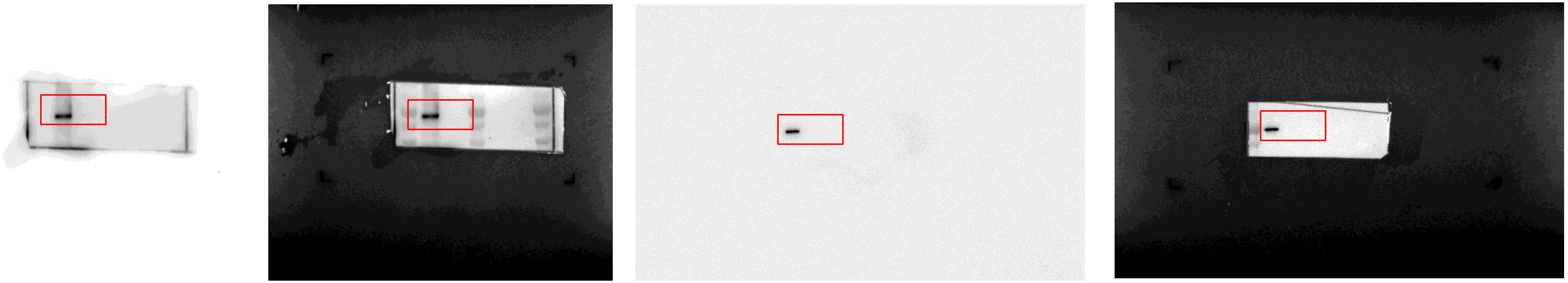

Corresponding to Figure 5D

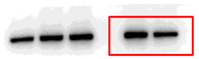

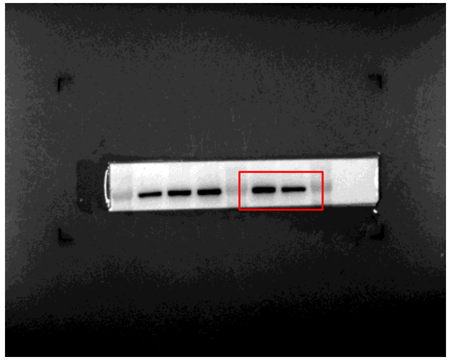

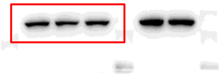

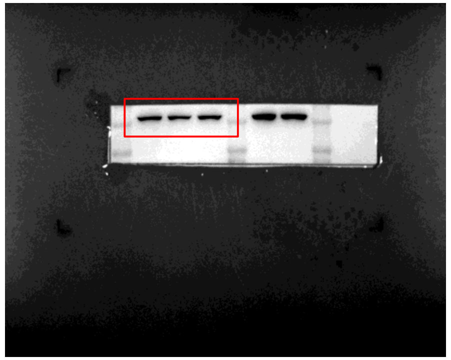

# Corresponding to Figure 5E

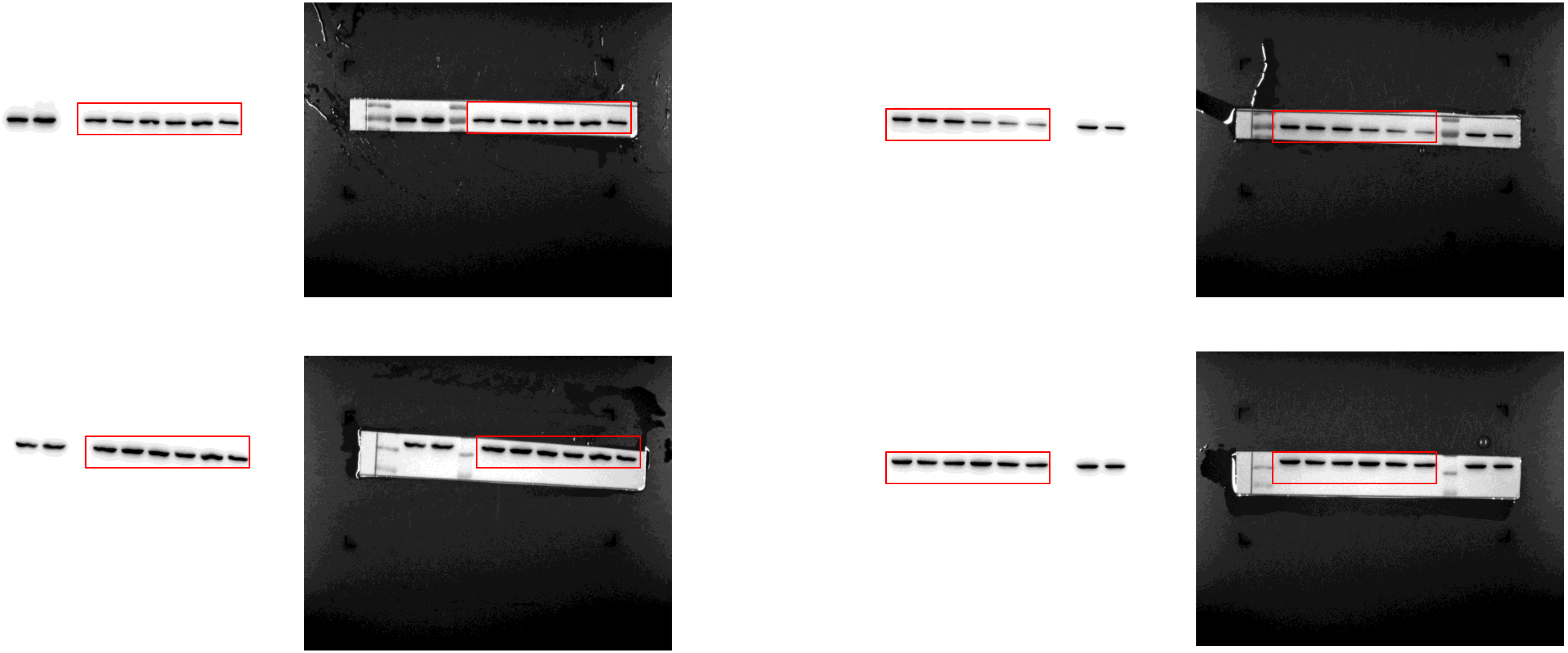

Corresponding to Figure 5F

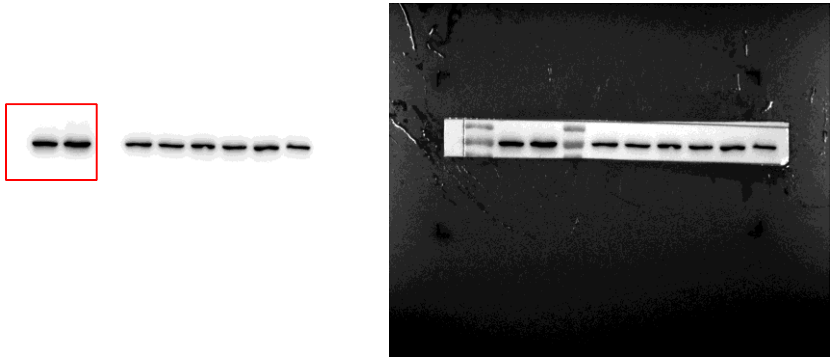

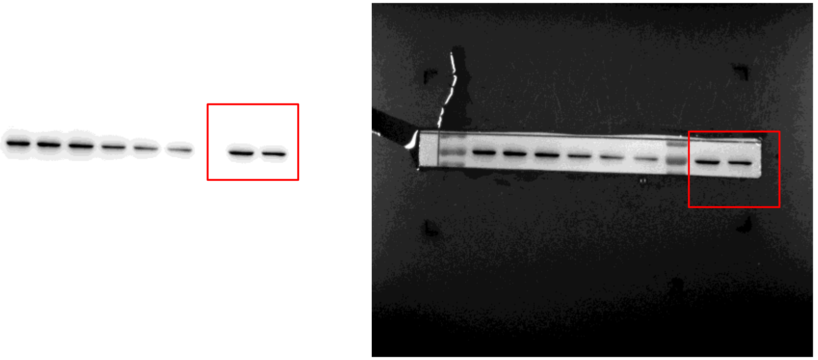

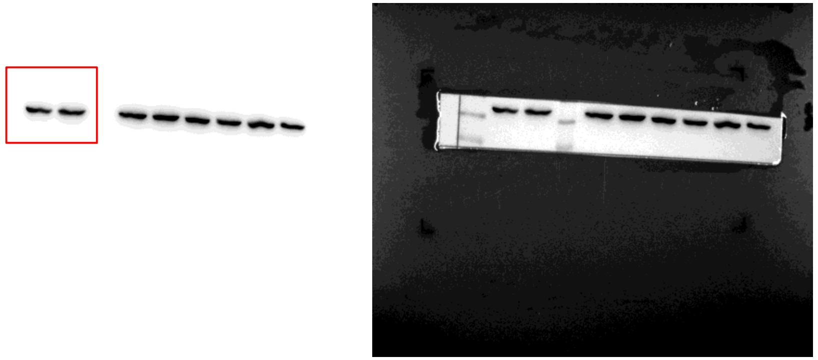

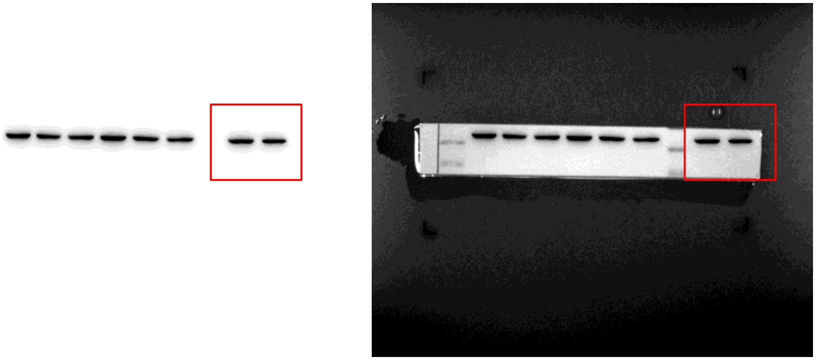

Corresponding to Figure 5G

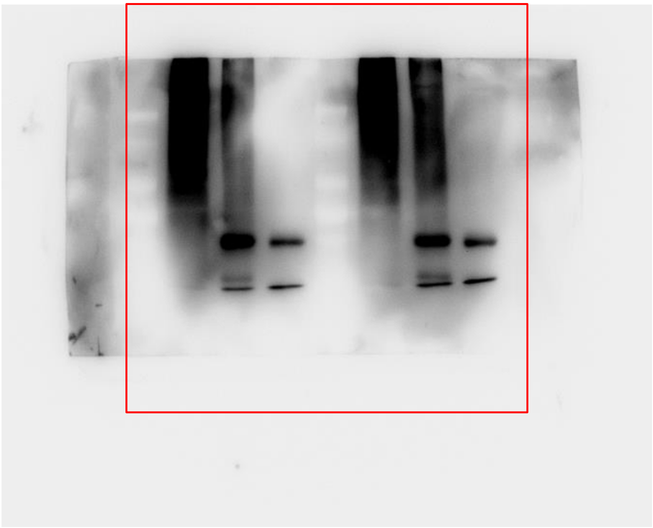

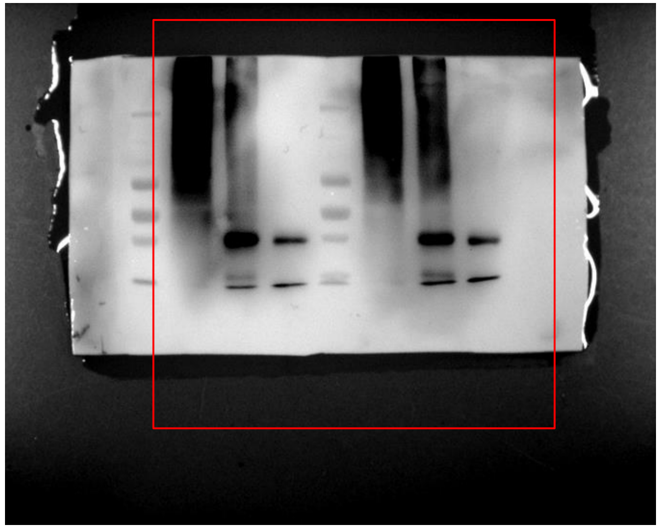

Corresponding to Figure 5I

TE-1

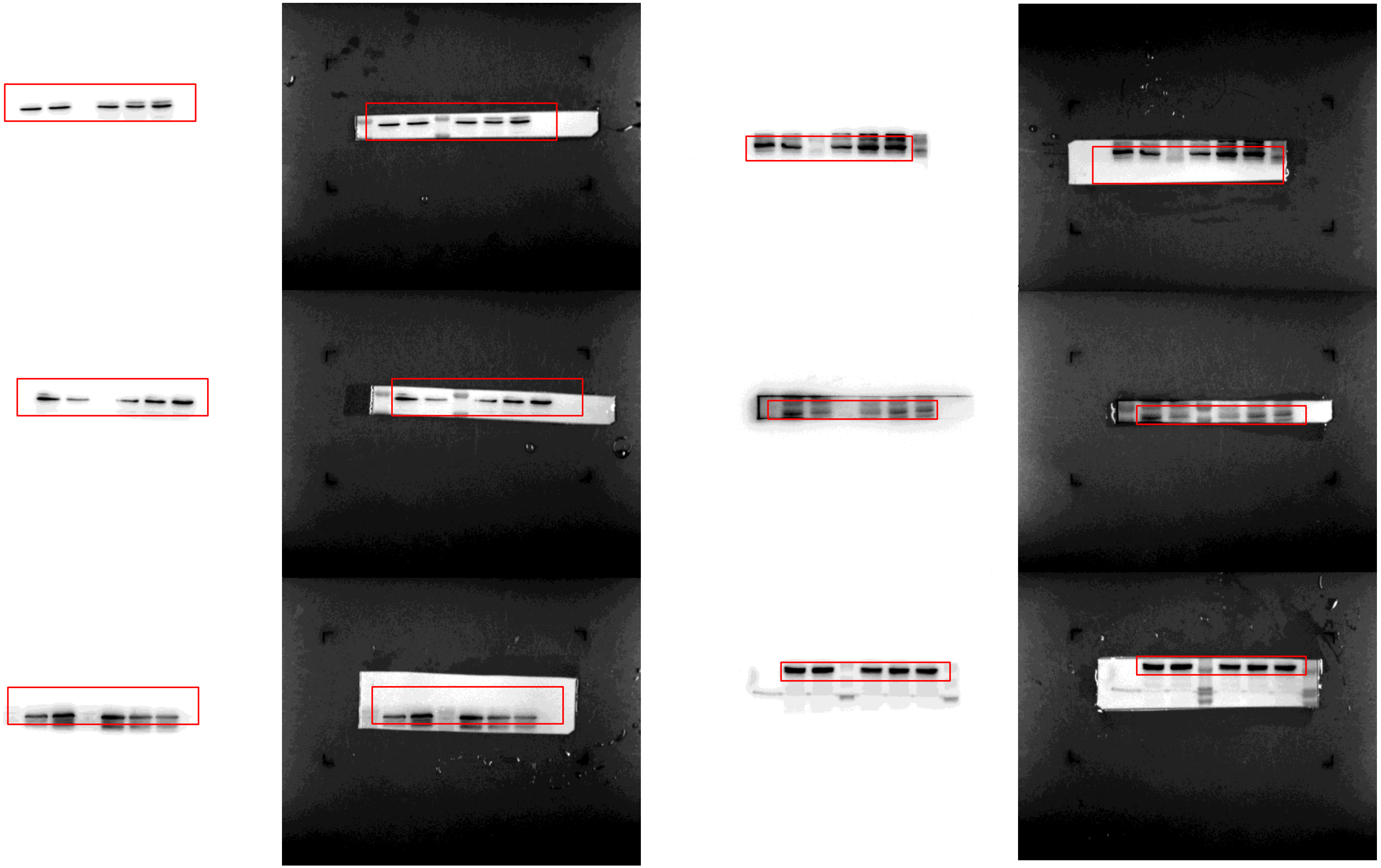

Corresponding to Figure 5I

KYSE-150

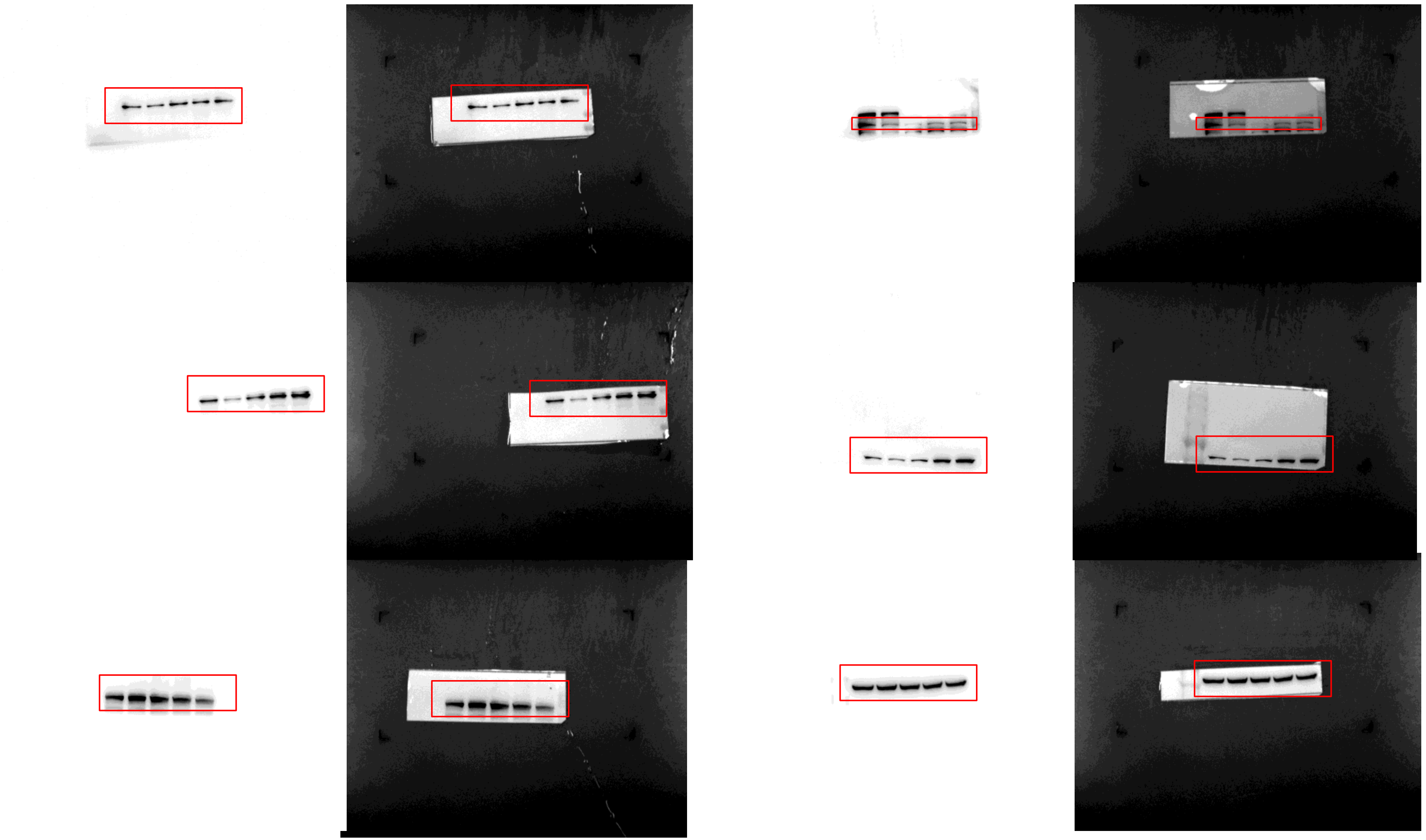

Corresponding to Figure 5J

TE-1

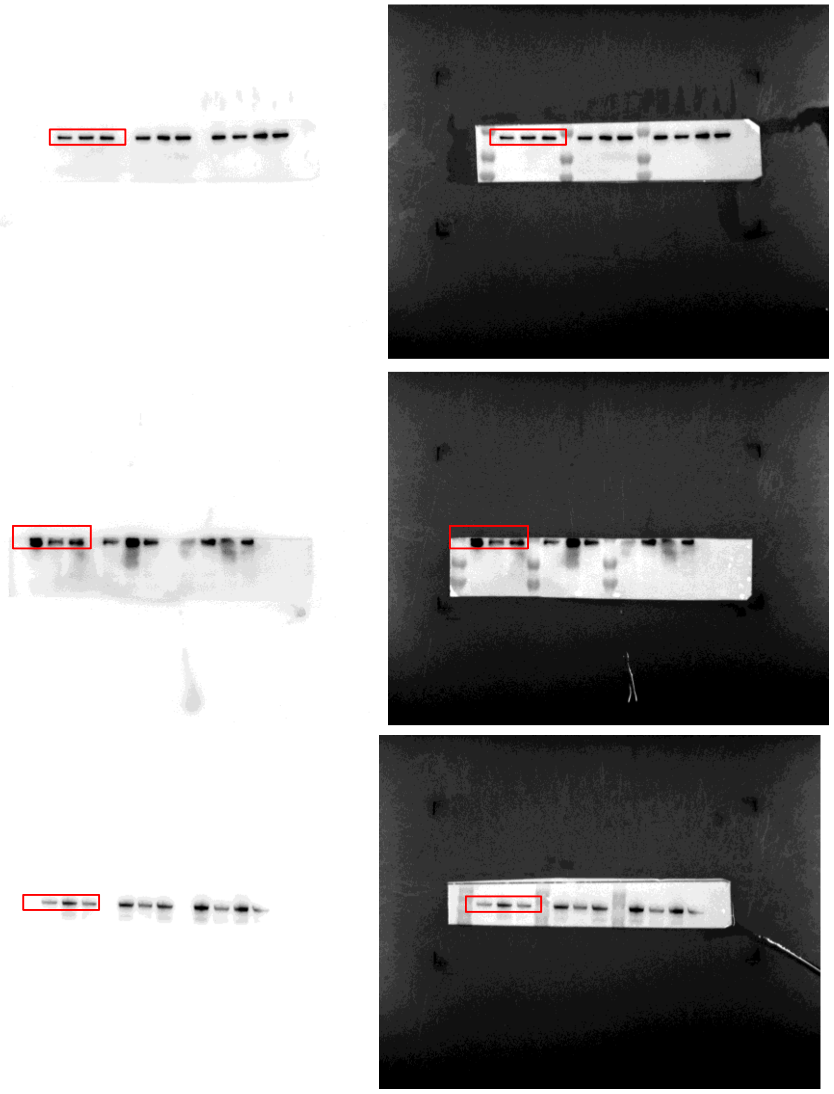

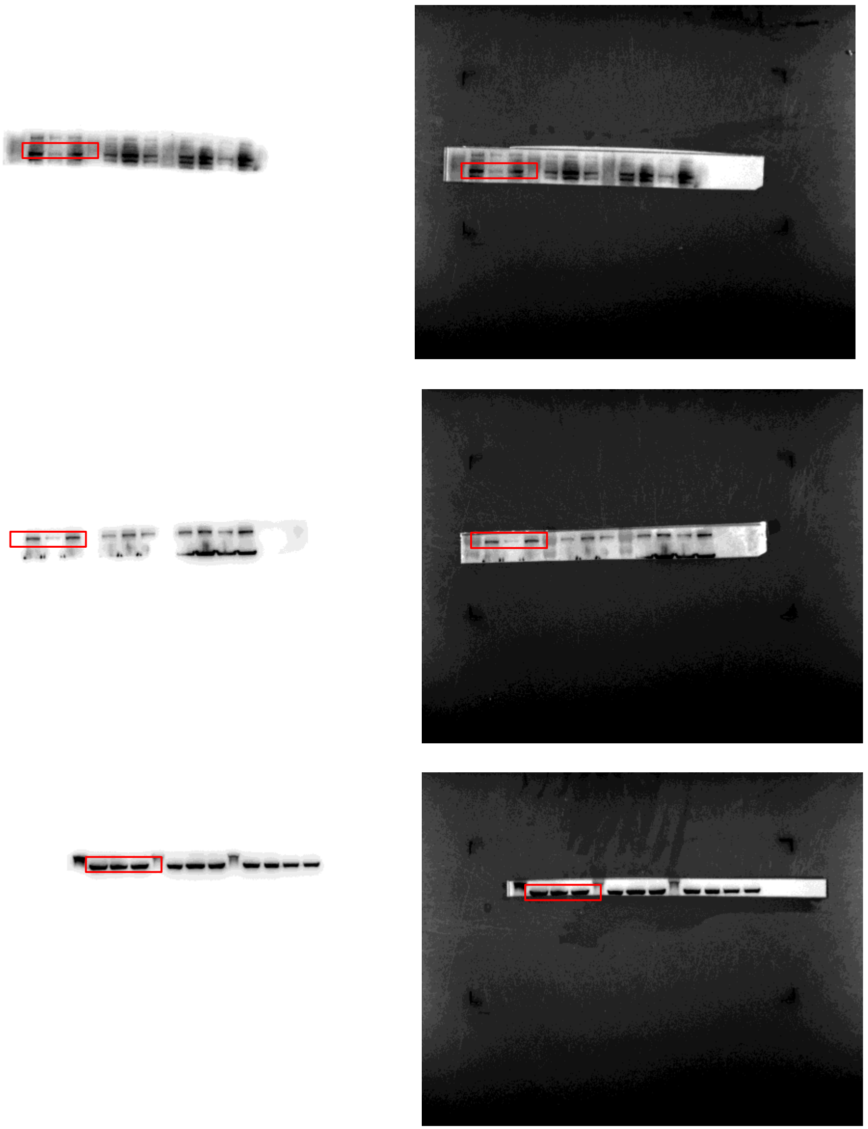

KYSE-150

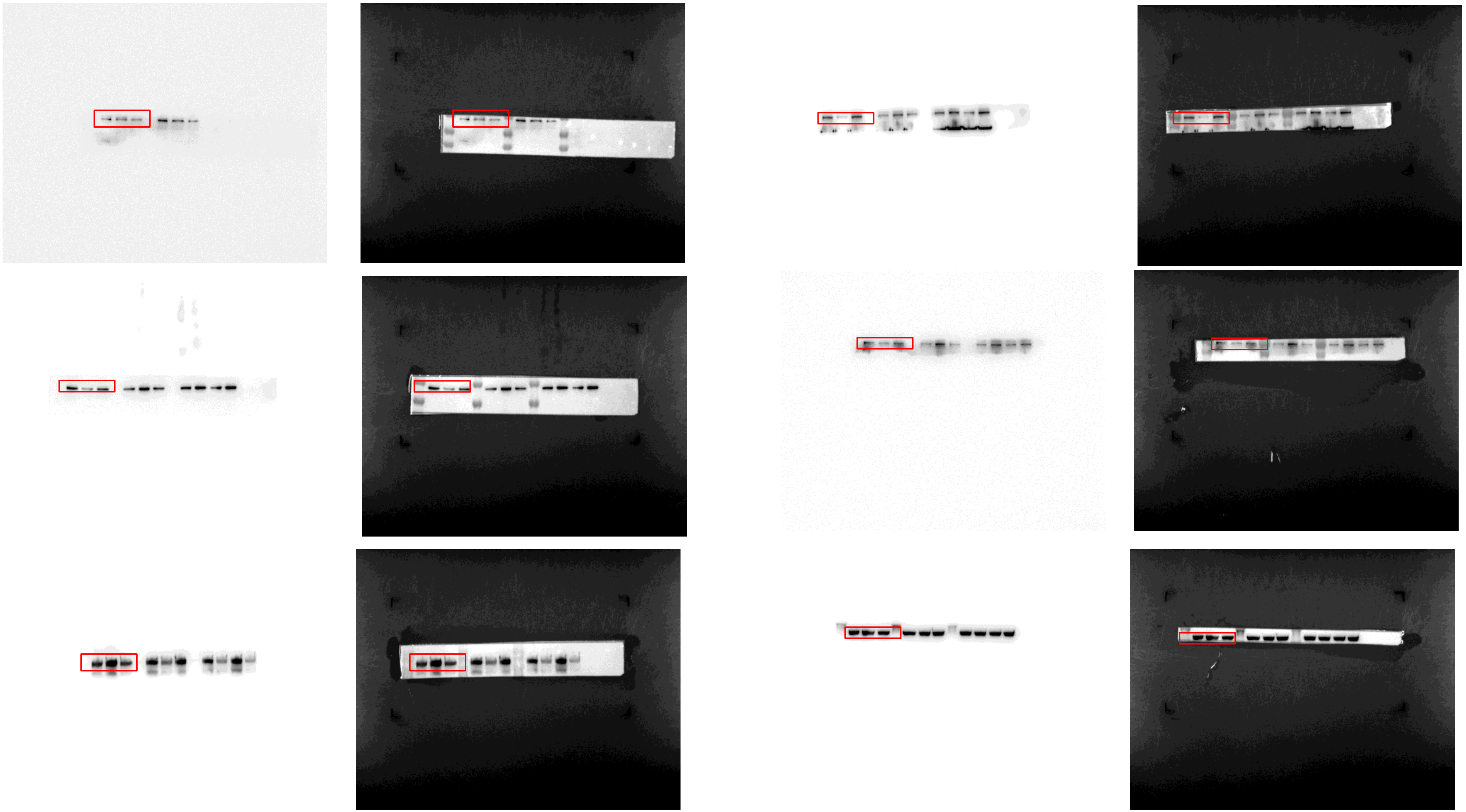

Corresponding to Supplemental figure 4A

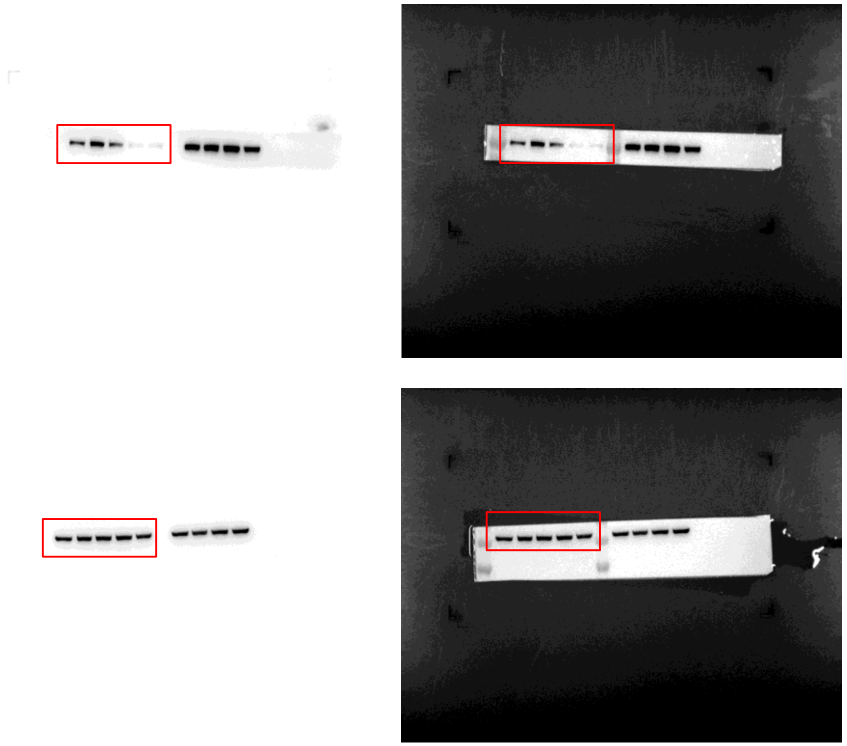

Corresponding to Supplemental figure 4B

TE-1

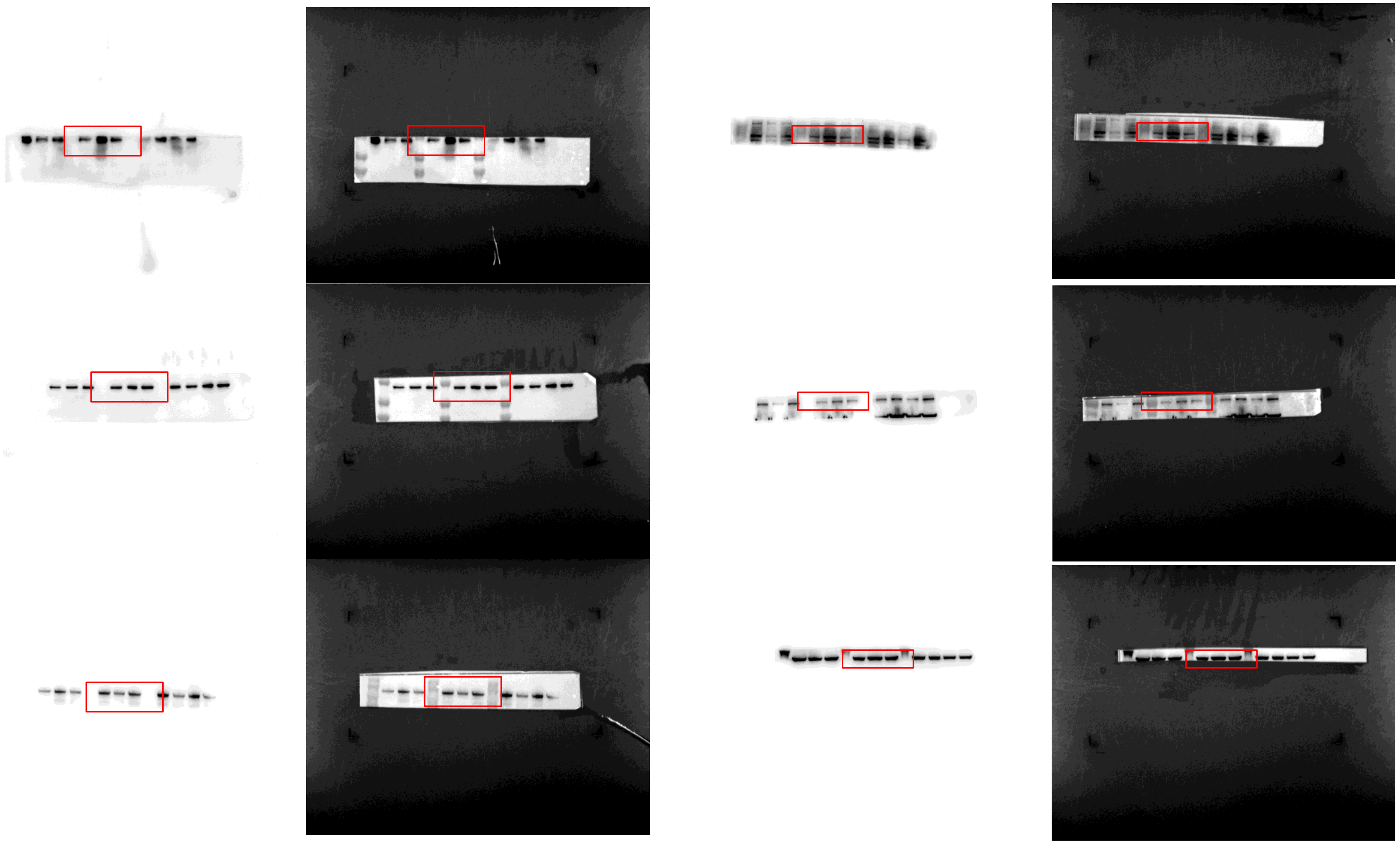

Corresponding to Supplemental figure 4B

KYSE-150

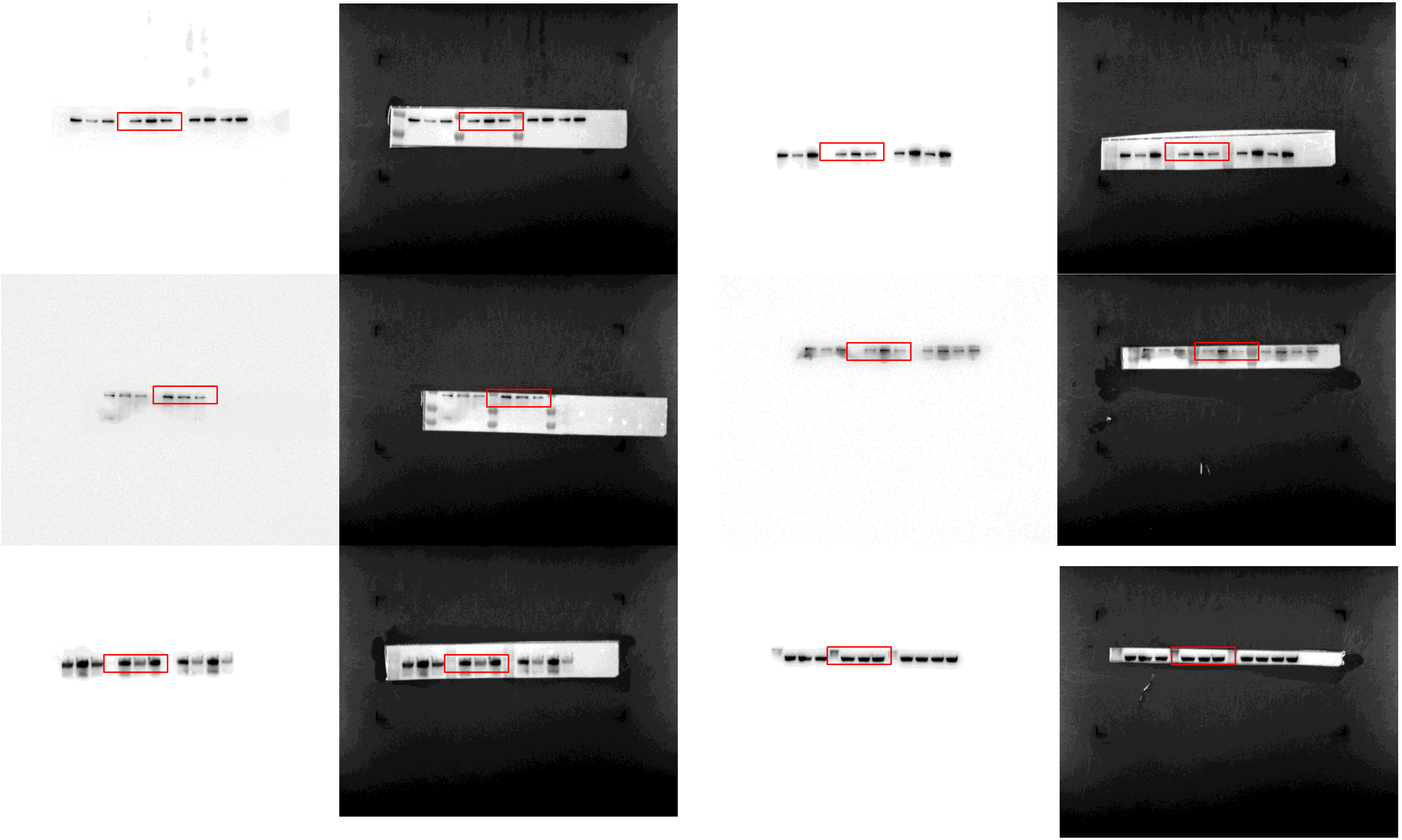

Corresponding to Supplemental figure 4C

TE-1

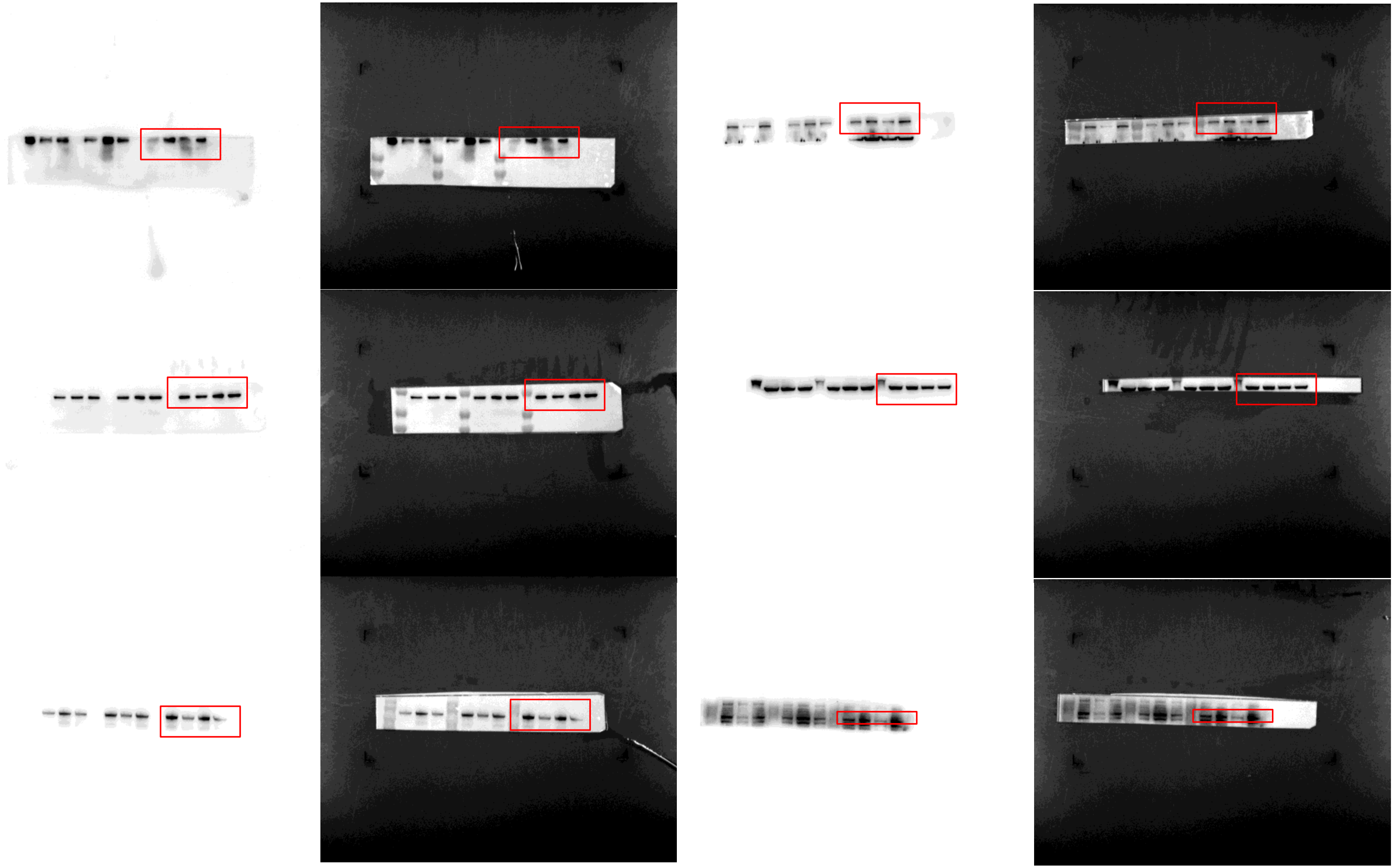

Corresponding to Supplemental figure 4C

KYSE-150

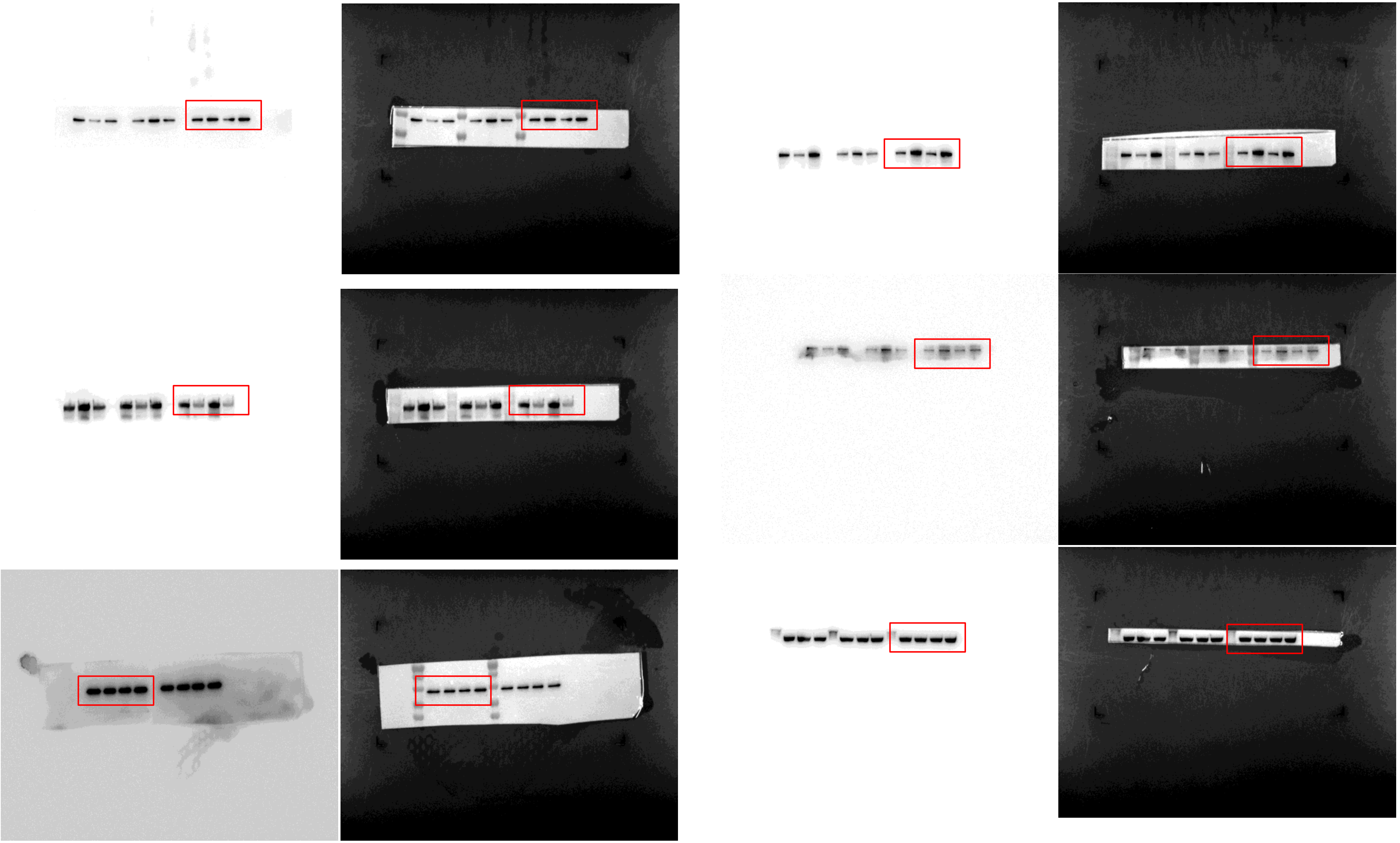

Supplement: Supplementary file 7 — Supplementary figure 5. Original western blots. [file 41419_2022_4818_MOESM7_ESM.pdf]
